# Chloroplast genome expansion by intron multiplication in the basal psychrophilic euglenoid *Eutreptiella pomquetensis*

**DOI:** 10.7717/peerj.3725

**Published:** 2017-08-25

**Authors:** Nadja Dabbagh, Matthew S. Bennett, Richard E. Triemer, Angelika Preisfeld

**Affiliations:** 1 Faculty of Mathematics and Natural Sciences, Zoology and Didactics of Biology, Bergische Universität Wuppertal, Wuppertal, Germany; 2 Department of Plant Biology, Michigan State University, East Lansing, MI, United States of America

**Keywords:** *Eutreptiella pomquetensis*, Introns, Twintrons, Genome structure, Chloroplast genome

## Abstract

**Background:**

Over the last few years multiple studies have been published showing a great diversity in size of chloroplast genomes (cpGenomes), and in the arrangement of gene clusters, in the Euglenales. However, while these genomes provided important insights into the evolution of cpGenomes across the Euglenales and within their genera, only two genomes were analyzed in regard to genomic variability between and within Euglenales and Eutreptiales. To better understand the dynamics of chloroplast genome evolution in early evolving Eutreptiales, this study focused on the cpGenome of *Eutreptiella pomquetensis*, and the spread and peculiarities of introns.

**Methods:**

The *Etl. pomquetensis* cpGenome was sequenced, annotated and afterwards examined in structure, size, gene order and intron content. These features were compared with other euglenoid cpGenomes as well as those of prasinophyte green algae, including *Pyramimonas parkeae*.

**Results and Discussion:**

With about 130,561 bp the chloroplast genome of *Etl. pomquetensis*, a basal taxon in the phototrophic euglenoids, was considerably larger than the two other Eutreptiales cpGenomes sequenced so far. Although the detected quadripartite structure resembled most green algae and plant chloroplast genomes, the gene content of the single copy regions in *Etl. pomquetensis* was completely different from those observed in green algae and plants. The gene composition of *Etl. pomquetensis* was extensively changed and turned out to be almost identical to other Eutreptiales and Euglenales, and not to *P. parkeae*. Furthermore, the cpGenome of *Etl. pomquetensis* was unexpectedly permeated by a high number of introns, which led to a substantially larger genome. The 51 identified introns of *Etl. pomquetensis* showed two major unique features: (i) more than half of the introns displayed a high level of pairwise identities; (ii) no group III introns could be identified in the protein coding genes. These findings support the hypothesis that group III introns are degenerated group II introns and evolved later.

## Introduction

Recent analyses of chloroplast genomes (cpGenomes) have been largely used to retrace evolutionary steps of phototrophic euglenoids. Members of the genera *Euglena*, *Monomorphina*, *Euglenaformis*, *Colacium*, *Strombomonas* and recently *Phacus* (Bennett, Wiegert & Triemer, 2012; Bennett, Wiegert & Triemer, 2014; Bennett & Triemer, 2015; Bennett, Shiu & Triemer, 2017; Dabbagh & Preisfeld, 2016; Gockel & Hachtel, 2000; Hallick et al., 1993; Kasiborski, Bennett & Linton, 2016; Pombert et al., 2012; Wiegert, Bennett & Triemer, 2013) cpGenomes of the ‘crown group’ Euglenales have been studied intensely. Overall aims were to tackle questions of relatedness, gene arrangement, synteny and genome size as well as possession and dispersal of introns. However, the knowledge on cpGenomes of the basal lineage, Eutreptiales, is comparatively low. The two known genomes were reported to show a smaller genome size and display only seven and 27 introns in *Eutreptiella gymnastica* and *Eutreptia viridis*, respectively (Hrdá et al., 2012; Wiegert, Bennett & Triemer, 2012). Fitting into a scheme of increasing intron quantity and genome size, the invasion of introns in euglenoids was assumed to have started with very low intron numbers and as a consequence small cpGenomes in Eutreptiales, which both increased during diversification of photosynthetic euglenoids (Bennett & Triemer, 2015; Hrdá et al., 2012; Thompson et al., 1995; Wiegert, Bennett & Triemer, 2012). This hypothesis was initially corroborated by the fact that in *Pyramimonas parkeae*, as the closest living relative of the euglenoid plastid, only one intron was detected (Turmel et al., 2009). However, this was later refuted by analysis of different lineages in the Euglenales, all of which presented species with large cpGenomes and more than 110 introns (both *E. gracilis* strains, *S. acuminata*, *C. vesiculosum*) in addition to small cpGenomes with low intron numbers like *M. aenigmatica* (Bennett & Triemer, 2015; Hallick et al., 1993; Pombert et al., 2012; Wiegert, Bennett & Triemer, 2013). Although it could be assumed that introns spread independently within the lineages, it was unknown whether a small or a large cpGenome was present when phototrophic euglenoids emerged and how (un)evenly these early introns were distributed in the Eutreptiales.

In the present study *Eutreptiella pomquetensis* (McLachlan, Seguel & Fritz) Marin & Melkonian in Marin et al. (2003) was analyzed as a member of the scarcely investigated Eutreptiales. It was originally isolated from shallow, cold, marine habitats and is the only known phototrophic euglenoid with four flagella (McLachlan, Seguel & Fritz, 1994). It was classified as an obligate psychrophilic species, which is an unusual characteristic for euglenoids, and worthy of investigation.

The Eutreptiales only consist of two genera, *Eutreptiella* da Cunha, with ten described species, and *Eutreptia* Perty, with eight known species. They are regarded as basal phototrophic euglenoids in aspects of morphology (Leander, Witek & Farmer, 2001; Leedale, 1967) as well as in molecular analyses and molecular studies combined with morphological characters (Linton et al., 1999; Linton et al., 2000; Marin et al., 2003; Preisfeld et al., 2001; Yamaguchi, Yubuki & Leander, 2012) and hence of particular interest, where the evolution of euglenoid chloroplasts is reflected upon. The capacity for photosynthesis in euglenoids was found to have originated with the acquisition of chloroplasts by a phagotrophic euglenoid via secondary endocytobiosis of a green alga in a marine environment, which is still unknown (Gibbs, 1978, Gibbs, 1981). Presumably, the donor was a relative of the partly obligatory psychrophilic genus *Pyramimonas* (Marin, 2004; Turmel et al., 2009).

Thus, it was our interest to investigate the psychrophilic *Eutreptiella pomquetensis* for two reasons: First, to compare this cpGenome with that of *P. parkeae* (Turmel et al., 2009), and the other two Eutreptiales (Hrdá et al., 2012; Wiegert, Bennett & Triemer, 2012) with regard to genome structure and size, intron number and propagation, and gene content as well as arrangement; second, to diminish the bias in taxon sampling in euglenoid cpGenomic analyses.

## Materials and Methods

### Growth, isolation, sequencing and assembly

*Eutreptiella pomquetensis* (McLachlan, Seguel & Fritz) Marin & Melkonian in Marin et al. (2003) strain CCMP 1491 cells were grown in modified L1-Si Medium (Guillard & Hargraves, 1993) with artificial seawater Sea-Pure (CaribSea, Inc. Fort Pierce, USA) at 2–4 °C with changing 3:3 light:dark cycle using ExoTerra Natural Light PT2190 (Hagen, Holm Germany).

Three-hundred mL of cell culture were harvested by centrifugation and submitted to cell cleaning and chloroplast isolation protocol as described in Dabbagh & Preisfeld (2016) with a slight change during sonication of cells. Purified cells were subjected to sonication twice for three seconds with the amplitude set at 50% and a pulse rate of 0.1 s (Bandelin Sonopuls HD 60; Bandelin, Berlin, Germany). The DNA was sequenced with 454 sequencing according to the GS FLX ++ chemistry Rapid Shotgun Library Preparation Method technology (Eurofins Genomics Ebersberg, Germany). In total 60,225 reads were produced in $ \frac{1}{4} $ segment of a full run with an average size of 608 bases. Automatic assembly of reads by Eurofins Genomics in Newbler (Roche, Basel, Switzerland) resulted in 668 contigs (N50 contig size was 1,157 bases).

### Annotation of the plastid genome

Using a BLASTn homology search (Altschul et al., 1990) the four largest contigs were identified as major parts of the plastid genome, and were subsequently linked by fill-in PCR from the end of each contig using whole genomic DNA. Contig 1 consists of 10,450 number of reads with an average coverage depth of 131.70, contig 2 of 3,918 number of reads with an average coverage depth of 129.90, contig 3 of 3,398 number of reads with an average coverage depth of 112.60. Contig 4 was identified as major part of the chloroplast rRNA operon and showed an average coverage depth of 211.30. The average depth is the mean read coverage and helps to identify repetitive parts of the chloroplast genome. Based on coverage depth of the ribosomal operon components (5S, 16S, 23S) compared to single copy protein coding genes, it appears that at least two copies of the operon are present on the genome. Closing the circle failed in spite of many different approaches using PCR experiments from *rpl*32 to *psa*C and further from each rRNA gene to *psa*C with specifically designed primers. Experiments to close the circle were performed with both a Long Range PCR Kit (Qiagen GmbH, Hilden, Germany) and a Long Amp Taq DNA Polymerase (New England BioLabs GmbH, Frankfurt am Main, Germany).

The final annotation of the chloroplast sequence was performed with Geneious 9 Pro (version 9.1.3, Kearse et al., 2012) with the option to translate the nucleotide sequence in all frames selected, and the “Genetic Code” was identified as “Bacterial”.

Protein coding genes were manually aligned in MEGA 7 (Tamura et al., 2011) against the nucleotide coding DNA sequences (CDS) from other photosynthetic euglenoids and prasinophyte representatives to determine exon-intron boundaries as well as start and stop of each gene. In all cases, a traditional methionine (ATG) start codon was preferred. CDS was verified by BLASTx, “Genetic code” set at “Bacteria and Archaea (11)” and Emboss Sixpack Sequence translation (EMBL- EBI 2015) “Codon Table” set at “Bacterial” and added to the annotation. The introns within protein coding genes were analyzed for the presence of potential twintrons as described in Bennett & Triemer (2015). This analysis was modified such that the 3′ motifs were established using a Python script instead of a manual search. The script browsed the homologous external introns for the conserved 3′ motifs (*abcdef* (3–8 nucleotides) *f*′*e*′*d*′*A*^∗^*c*′*b*′*a*′ (four nucleotides)). Afterwards, all 51 introns were searched for the conserved 5′ insertion sequence GUGYG. RNA secondary structure for group II introns was created by RNA folding via Mfold web server using default settings (Zuker, 2003), manually optimized and illustrated with the PseudoViewer web application (Byun & Han, 2006). For *roa*A a pairwise sequence comparison of the amino acid sequence of *E. gracilis* with a putative amino acid sequence of *Etl. pomquetensis* was performed using Exonerate 2.4.0 by Slater & Birney (2005) to reveal intron boundaries and start/ stop of the searched gene.

tRNAscan-SE 1.21 (Schattner, Brooks & Lowe, 2005), with the default settings and the source given as mito/chloroplast, was used to identify tRNAs. Uncharacterized open reading frames (ORFs) were identified with ORF finder within Geneious, with the genetic code set to bacterial. Only ORFs which were at least 300 bp, did not overlap with the coding region of another gene, and lacked BLASTp evidence (default settings) for being a previously identified chloroplast protein-coding gene were included in the annotation. ORFs were named according to the number of amino acids in the coding region. To evaluate the proportion of short repeated sequences the variable number of tandem repeats was scanned with the online version of REPuter (Kurtz et al., 2001) under the same settings as described in Bennett & Triemer (2015) and with Tandem Repeats Finder, with the option “Basic”, using default parameters (Benson, 1999).

The start/stop areas of the 16S and 23S rRNA genes were identified using RNAmmer 1.2 (Lagesen et al., 2007), with “Bacteria” chosen as the sequence kingdom of origin. The 5S rRNA start/stop regions were identified using Rfam 12.1 Sequence Search (Burge et al., 2013). The number of rRNA operons flanked by the protein-coding genes *rpo*C2 and *psb*A were confirmed using PCR. One further rRNA operon was identified by PCR experiments next to the protein coding gene *rpl*32 by long range PCR. To verify the exact sequence a Long Range PCR was performed with primers (forward 5′ -AGAGTTTGATCCTGGCTCAG- 3′; reverse 5′-TGCTTCCATACACTTTTACGCATA- 3′) from the beginning of the 16S to the *rpl*32 gene. Primers were created manually by Primer3Plus (Untergasser et al., 2012) based on the nucleotide sequence. The PCR product (5,080 bp) was purified and used as DNA matrix for further PCRs to determine the sequence of the rRNA genes and the noncoding regions in between. The number of rRNA operons next to the *rpl*32 gene was performed using long-range PCR. The long-range PCR approach to measure the copies of the RNA operon yielded only one product.

Synteny between the cpGenomes of all three sequenced Eutreptiales was determined using Mauve (Darling et al., 2004), as a plugin for Geneious, with the alignment algorithm set as progressive Mauve. Each genome was displayed as a linear sequence with blocks representing a homologous gene cluster. In the Mauve alignment the repeat regions of rRNA were not included because Mauve will not align repeat regions which have multiple matches on both genomes. The circular genome map was created using GenomeVx (Conant & Wolfe, 2008).

## Results and Discussion

### General genome analyses

**Figure 1:**
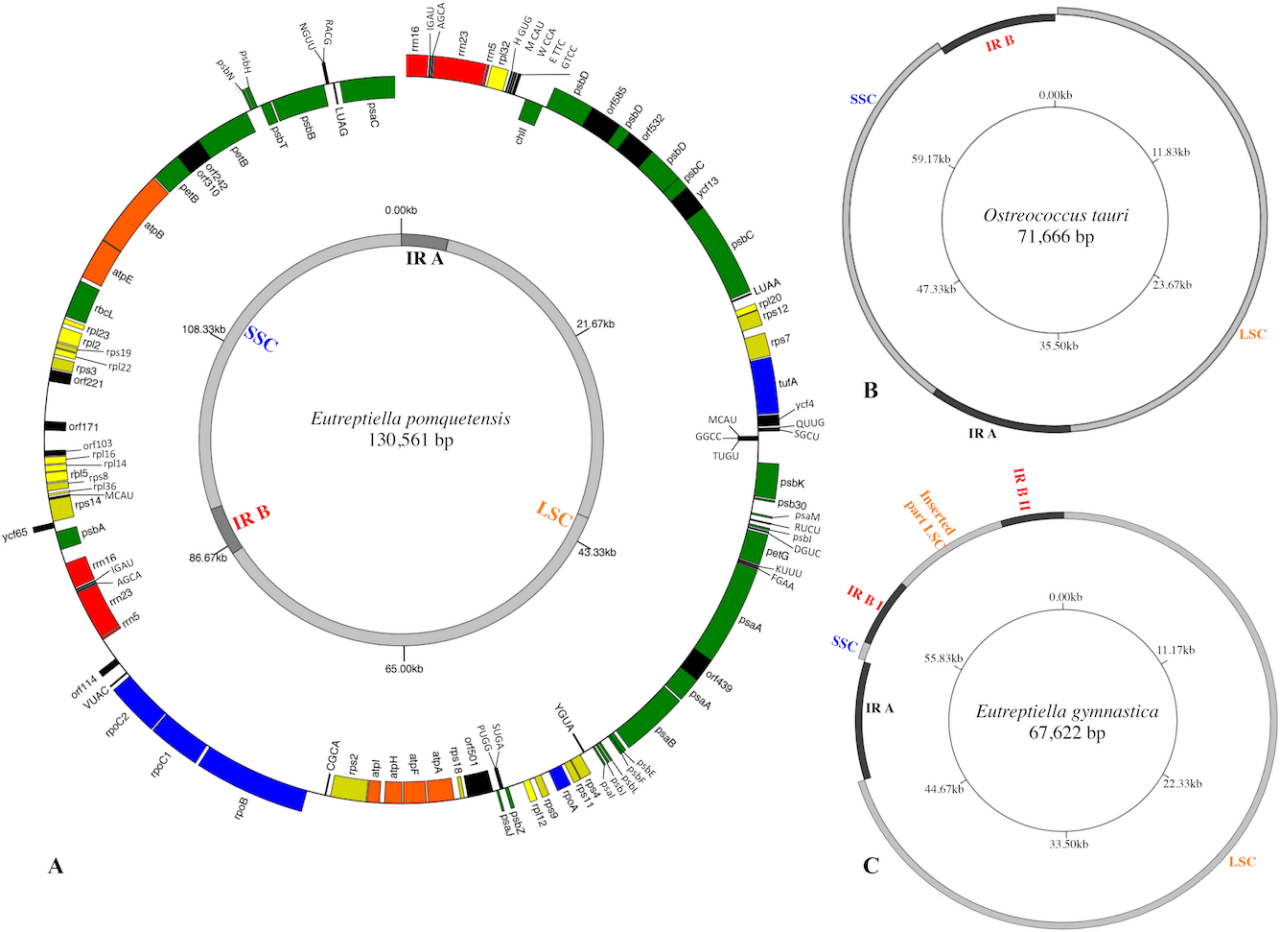
Gene maps of chloroplast genomes. (A) Map of the plastid genome of *Eutreptiella pomquetensis*. Boxes of different colors represent genes of similar functional groups: red, ribosomal rRNAs; green, photosystem/photosynthesis genes; yellow, ribosomal proteins (*rpl*, *rps*); orange, *atp* genes; blue: transcription/translation- related genes (*rpo*, *tuf*A); black, conserved hypothetical proteins (*ycf*), open reading frames (ORF), tRNAs. Boxes are proportional to their sequence length. Outer ring: Genes on the outside of the circle are considered on the positive strand, genes inside the circle on the negative strand. Inner circle shows the large single copy region (LSC) and short single copy region (SSC) in light grey and inverted repeats (IR) in dark grey. (B–C) Simplified maps of the plastid genomes of *Ostreococcus tauri* (B) and *Eutreptiella gymnastica* (C) to demonstrate similarities and differences of genome structures. Copies of the IR sequences are represented in dark grey and LSC and SSC in light grey.

The cpGenome of *Etl. pomquetensis* is presented as an incomplete circle, because attempts to close the gap between the 16S rRNA gene and the protein coding gene *psa*C were unsuccessful, even with long range approaches. Thus, the cpGenome contained at least 130,561 bp, which is twice the size of *Eutreptiella gymnastica* with 67,622 bp (Hrdá et al., 2012) and *Eutreptia viridis* with 65,523 bp (Wiegert, Bennett & Triemer, 2012). The new cpGenome resembled the members of the Euglenales *E. gracilis var. bacillaris* (132,034 bp) and *C. vesiculosum* (128,892 bp, Table S1) in size. The content of genes was similar to those of other phototrophic euglenoids and reduced as compared to *P. parkeae* (Turmel et al., 2009) or *Ostreococcus tauri* (Robbens et al., 2007). The organization of the whole genome, however, resembled those of higher plants and algae (Cattolico et al., 2008; Lemieux, Otis & Turmel, 2007; Ravi et al., 2008; Robbens et al., 2007; Turmel et al., 2009) more than other euglenoids. The genome was composed of a large single copy region (LSC 80,941 bp), a small single copy region (SSC 39,856 bp) and two inverted repeats (IR) containing the rRNA genes in a way similar to *O. tauri,* but different in gene content (Figs. 1A & 1B). In the cpGenome of *P. parkeae*, the putative chloroplast donor for euglenoids, the organization is very much alike, but lacks the 5S rRNA in both inverted repeats. However, the possibility of non-recognition of the sequence as described by Turmel et al. (2009) still has to be considered. The fact that one operon was localized on the positive and one on the negative strand points at another similarity between the green algae *P. parkeae, O.tauri* and *Etl. pomquetensi* s. In the close relative *Etl. gymnastica,* the rRNA operon consisted of two incomplete copies, without a 5S rRNA, as in *P. parkeae*, but additionally one operon was divided into two parts separated by parts of the LSC (Hrdá et al., 2012, Fig. 1C). The G+C base composition of 35.1% again resembled that of *Etl. gymnastica* and *P. parkeae* and was higher than that of *Et. viridis* with 28.6% (Table S1).

### Analysis of gene content and arrangement

In total, 94 genes were identified and annotated in the cpGenome of *Etl. pomquetensis*, including 60 protein coding genes, two complete copies of the rRNA operon and 28 tRNAs (Fig. 1A). Alignments and analysis of protein coding genes indicated that the coding regions were more similar to those of *P. parkeae* than to those of the *Euglena* clade. For example, a pairwise comparison between *psb*D coding regions from *P. parkeae* and *Etl. pomquetensis* pointed out an 84.4% identity at the nucleotide level, whereas the same region from *E. gracilis* and *Etl. pomquetensis* showed only an 80.5% identity making the resemblance of *Etl. pomquetensis* to *P. parkeae* more apparent. Traditional methionine start codons (ATG) were found for each protein coding gene, except *rpo*A, where an alternative start codon (ATA) was accepted. Four protein coding genes were annotated with alternative start codons in *Etl. gymnastica* and *Et. viridis* (Table 1; Hrdá et al., 2012; Wiegert, Bennett & Triemer, 2012) and three in *P. parkeae* (Turmel et al., 2009). The number of the protein coding genes (60) was most similar to *Etl. gymnastica* (59), where *psa*I was missing. *Et. viridis* lacked *psa*M, *ycf*12 (*psb*30) and *ycf*65 and hence counted only 57 protein coding genes. No *mat*5 or *mat*2 genes have been identified in *Etl. pomquetensis*, but *mat*1 (*ycf*13) was detected, as was expected from results in other Eutreptiales (Hrdá et al., 2012; Wiegert, Bennett & Triemer, 2012). Just like *Et. viridis* (Wiegert, Bennett & Triemer, 2012), *Etl. pomquetensis* also lacked the common land plant chloroplast genes *rpl*33, *inf*A, *clp*P, *frx*B, *ndh*A-K, *pet*A, *pet*D, *psb*M, *rps*15 and *rps*16.

**Table 1:** Alternative start codons usage in protein coding genes of cpGenomes of Eutreptiales and *Pyramimonas parkeae* (Chlorophyta).

	Alternative start codons:			
	*Etl.pomquetensis*	*Etl. gymnastica*	*Et. viridis*	*P. parkeae*
Total number	1	4	4	3
Gene/start	*rpo*A (ATA)	*rpo*A (TTG)	*psa*I (ATT)	rps11 (GTG)
		*psb*C (TAT)	*rps*11 (ATT)	*rpo*A (GTG)
		*ycf*13 (GTG)	*atp*E (ATT)	*rps*18 (GTG)
		*atp*F (TTG)	*pet*B (GTG)	

**Figure 2:**
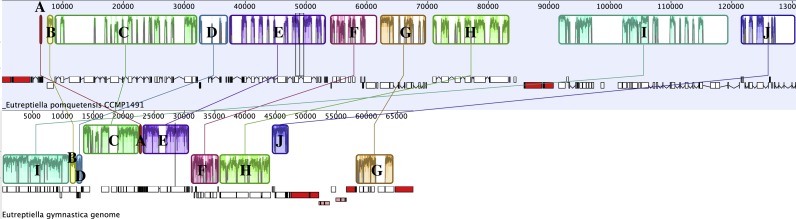
Progressive Mauve analysis comparing the cpGenomes of *Etl. pomquetensis* and *Etl. gymnastica*. Each box represents a cluster of homologous genes with *Eutreptiella pomquetensis* as the reference genome. Like blocks are labelled by letters A–J. See Table 4 for a list of genes contained in each block. In the Mauve alignment the repeat regions of rRNA were not included, because Mauve will not align repeat regions, which have multiple matches on both genomes.

Progressive Mauve was used to analyze related chloroplast genomes (Darling et al., 2004). A comparison of *Etl. pomquetensis* and *Etl. gymnastica* gene content and arrangement identified 10 conserved gene clusters (Fig. 2, Table 2). Although gene content was similar in the two studied *Eutreptiella* species, the gene clusters showed significant rearrangements in position and strand orientation between *Etl. gymnastica* and *Etl. pomquetensis*. Block I was the largest in *Etl. pomquetensis*, included 18 genes, and was more than 19 kb long. The clusters themselves showed that extensive rearrangements occurred between *Etl. gymnastica* and *Etl. pomquetensis*. This lack of synteny was surprising, because high intrageneric variability between other taxa had not been noted so far. For example, a comparison between *M. aenigmatica* and *M. parapyrum* or *E. gracilis* and *E. viridis* cpGenomes revealed only one and two blocks, respectively. But, although *Etl. gymnastica* and *Etl. pomquetensis* are described as belonging to one genus, the evolutionary distance between euglenoid taxa is usually relatively high and makes differences probable. On the other hand, *Etl. pomquetensis* lives under psychrophilic conditions, whereas *Etl. gymnastica* lives under moderate marine conditions, which means that the environmental pressure is varying.

**Table 2:** Gene clusters resulting from Progressive Mauve cpGenome analysis of the two *Eutreptiella* species. Gene clusters (blocks) are labelled with letters (A–J) and relevant genes listed. Bars in blocks C, H, I, J mark positions of a second Progressive Mauve analysis of the three Eutreptiales, where blocks are divided (see Fig. S1).

Block	Gene Clusters
A	tRNA-His, tRNA-Met, tRNA-Trp, tRNA-Glu, tRNA-Gly
B	*chl*I
C	*psb*D *psb*C tRNA-Leu **/***rpl*20 *rps*12 *rps*7 *tuf*A ycf4 tRNA-Gln tRNA-Ser
D	tRNA-Arg *psa*M *psb*30 (syn. *ycf*12) psbK tRNA-Thr tRNA-Gly tRNA-Met
E	*psb* I tRNA-Asp *pet*G tRNA-Lys tRNA-Phe *psa*A *psa*B *psb*E *psb*F *psb*L *psbJ*
F	*rps*18 *psa*J tRNA-Pro tRNA-Ser *psb*Z *rpl*12 *rps*9 *rpo*A *rps*11 *rps*4 tRNA-Tyr
G	tRNA-Cys *rps*2 *atp*I *atp*H *atp*F *atp*A
H	tRNA-Val **/***rpo*C2 *rpo*C1 *rpo*B
I	*pet*B *atp*B *atp*E **/***rbc*L *rpl*23 *rpl*2 *rps*19 *rpl*22 *rps*3 *rpl*16 *rpl*14 *rpl*5 *rps*8 *rpl*36 tRNA-Met *rps*14 ycf65 *psb*A
J	*psb*N *psb*H *psb*T *psb*B tRNA-Asn tRNA-Arg tRNA-Leu **/***psa*C

The noted difference in gene density between *Etl. pomquetensis* and *Etl. gymnastica* was not only due to an increase of introns from seven introns in *Etl. gymnastica* (total amount of intron space 6,893 bp) to 51 introns in *Etl. pomquetensis* (total amount of intron space 52,999 bp), but additionally to an increased intergenic space in *Etl. pomquetensis*. The intergenic space of *Etl. pomquetensis* comprised more than 23 kb, which was more than twice in that of *Etl. gymnastica*. While most of the blocks in *Etl. gymnastica* were quite compact with little intergenic or intron space in blocks C, E and G, all of the identified clusters showed heavily fragmented blocks in *Etl. pomquetensis*, except A and B (Fig. 2).

A second Mauve analysis of *Etl. pomquetensis* and the two other basal phototrophic Eutreptiales *Et. viridis* and *Etl. gymnastica* identified 14 conserved gene clusters (Fig. S1). The gene order within the clusters was mostly conserved and equal to the ten clusters found in the previous analysis. However, four gene clusters were further divided into two clusters each (Table 2, bar in blocks C, H, I, J).

Three additional Mauve analyses using *Etl. pomquetensis* identified 31 clusters with *P. parkeae*, 26 with *P. provasolii*, and 21 with *O. tauri* (Fig. S2). A comparison of the Mauve analyses found more homologous regions between *Etl. pomquetensis* and the other Eutreptiales than with the prasinophytes (the group containing the putative chloroplast donor). As the phototrophic euglenoids have a reduced amount of protein coding genes in contrast to the green algae, this high number of clusters was expected.

### Open reading frames

Ten uncharacterized open reading frames (ORFs) were found in *Etl. pomquetensis*. A BLASTp analysis was performed against the NCBI nonredundant protein sequences (nr) database to determine whether any of the ORFs had functional similarity to previously sequenced genes (Table 3). The *psb*D gene of *Etl. pomquetensis* contained two ORFs (*orf*585 and *orf*532). The intron encoded *orf*585 of *Etl. pomquetensis psb*D I2 shared strong similarity with the *orf*583 of *atp*B I1 in the chloroplast genome of *Pycnococcus provasolii* (Turmel et al., 2009), with an *e*-value of 0.0. Turmel et al. (2009) determined that the *Pycnococcus* and *Ostreococcus* intron ORFs share strong similarity with each other, and for example, also with *mat*4 in *Euglena myxocylindracea* (Turmel et al., 2009). The open reading frames *orf*171 and *orf*103 next to the *rpl*16 gene showed weak similarity to the *roa*A gene annotated in some Euglenales chloroplast genomes. However, in either case the best match is reported for putative reverse transcriptase and intron maturase. Further, exonerate 2.4.0 (Slater & Birney, 2005) and a manual alignment were performed to evaluate if the two ORFs were part of the *roa*A gene. Neither of these methods yielded clear results, and no exact exon-intron boundaries or start/ stop regions could be identified. Additionally, RT-PCR experiments for detecting a putative intron between *orf*103 and *orf*171 failed, indicating that these ORFs may not have a true function *in vivo*.

**Table 3:** Open Reading Frames. BLASTp analysis of ten uncharacterized ORFs in the *Eutreptiella pomquetensis* cpGenome against NCBI nonredundant protein sequences (nr) database. For each ORF the best match is reported.

ORF	Accession number	Best BLASTp match		
		Organism	Product	*E*-value
585	YP_002600812.1	*Pycnococcus provasolii*	putative reverse transcriptase and intron maturase	0.0
532	WP_041039849.1	*Tolypothrix campylonemoides*	group II intron reverse transcriptase/maturase	3*e* − 57
439	YP_009306333.1	*Caulerpa cliftonii*	hypothetical protein	2*e* − 39
501	WP_050045085.1	*Tolypothrix bouteillei*	group II intron reverse transcriptase/maturase	4*e* − 65
114	−	−	no significant similarity found	−
310	BAM65725.1	*Helminthostachys zeylanica*	maturase K	9*e* − 14
242	WP_061793822.1	*Bacillus firmus*	hypothetical protein	0.14
221	AOC61650.1	*Gloeotilopsis planctonica*	putative reverse transcriptase and intron maturase	5*e* − 35
171^a^	AOC61481.1	*Gloeotilopsis sarcinoidea*	putative reverse transcriptase and intron maturase	3*e* − 12
103^a^	AOC61650.1	*Gloeotilopsis planctonica*	putative reverse transcriptase and intron maturase	7*e* − 09

**Notes.**

a maybe *roa*A.

**Table 4:** Features of the presumed ancestral *psb*C twintron in all cpGenomes of phototrophic euglenoids.

	Intron containing *mat*1	*psb*C total Intron length (bp)	length *mat*1 (bp)	*psb*C intron length without *mat*1 (bp)
*E. gracilis*	I4	1,605	1,377	228
*E. gracilis var. bacillaris*	I4	1,605	1,377	228
*E. viridis*	I2	1,612	1,359	258
*E. viridis epitype*	I2	1,617	1,359	258
*E. mutabilis*	I2	3,406	3,149	257
*Era. anabaena*	I2	1,945	1,683	262
*M. parapyrum*	I2	1,613	1,338	275
*M. aenigmatica*	I2	1,618	1,389	229
*Cr. skujae*	I3	1,629	1,362	267
*S. acuminata*	I2	1,686	1,371	315
*T. volvocina*	I2	2,534	1,672	862
*C. vesiculosum*	^a^	2,742		
*Efs. proxima*	I3	3,349	2,669	680
*P. orbicularis*	I1	1,716	1,533	183
*Et. viridis*	I1	4,350	3,609	741
*Etl. gymnastica*	I1	1,778	1,137	641
*Etl. pomquetensis*	I1	2,580	1,389	1,191

**Notes.**

a annotation mistake.

There is no evidence of a VNTR (variable number of tandem repeat) sequence, though this could be a result of our inability to circularize the genome.

### Intron sequence similarity

Twenty-three out of the 60 protein-coding genes contained one or more introns, resulting in a total of 51 introns with likely twintrons measured as one insertion site. *psa*A contained the highest count with six introns (Table S1). The number of introns revealed, is twice as high as in *Et. viridis* (27), nearly eight times higher than found in *Etl. gymnastica* (7), and consequently constitutes the highest intron number known in the Eutreptiales (Hrdá et al., 2012; Pombert et al., 2012; Wiegert, Bennett & Triemer, 2012). Upon closer inspection of the intron sequences, we discovered 90% pairwise identities in introns of different genes in *Etl. pomquetensis*.

Therefore, and to gather information on the relatedness of the introns in basal euglenoids, we aligned all intron sequences and detected 28 introns (773–1,578 bp, Table S2 marked bold) in *Etl. pomquetensis* with pairwise identities of 87.4% and identical 5′-GTGCG boundaries typical for group II introns. Since group II introns in euglenoids are short for group II intron membership and usually do not show high sequence similarities, except in bounding regions, the strongly conserved GAAA terminal loop and portions of the domain V stem and, if present, in maturases (Michel & Ferat, 1995; Thompson et al., 1997) it was surprising to discover pairwise identities of about 90% in introns of different genes in *Etl. pomquetensis*. Moreover, 3′ boundaries always showed matching ACGTTCAT motifs (except for *pet*G I1 and *psa*C I2) with the presumed “branch-point” *A for splicing at position eight in domain VI, where the first transesterification takes place (Lambowitz & Belfort, 2015). The last two nucleotides AY represent the typical conserved ending for group II-introns (Lambowitz & Belfort, 2015). As expected, domain V, known to play a catalytic role in intron excision, showed a highly conserved secondary structure (Kelchner, 2002; Michel & Ferat, 1995; Thompson et al., 1997; Toor, Hausner & Zimmerly, 2001). The 28 introns scrutinized, except for *pet*G I1 and *psa*C I2 (Table S2 marked bold), showed a highly conserved domain V with 24 out of 34 nucleotides identical. Beside the fact that three base pairs (5′- …AGC …GUU…-3′) near the base of the stem were completely identical (Fig. S3), the secondary structure was unambiguously the same as the secondary structure of group IIB introns predicted by Kelchner (2002). Also of interest was that more than half out of the 51 nucleotides forming the stem and loop of domain VI were identical and resulted in the same secondary structure (Fig. S3).

**Figure 3:**
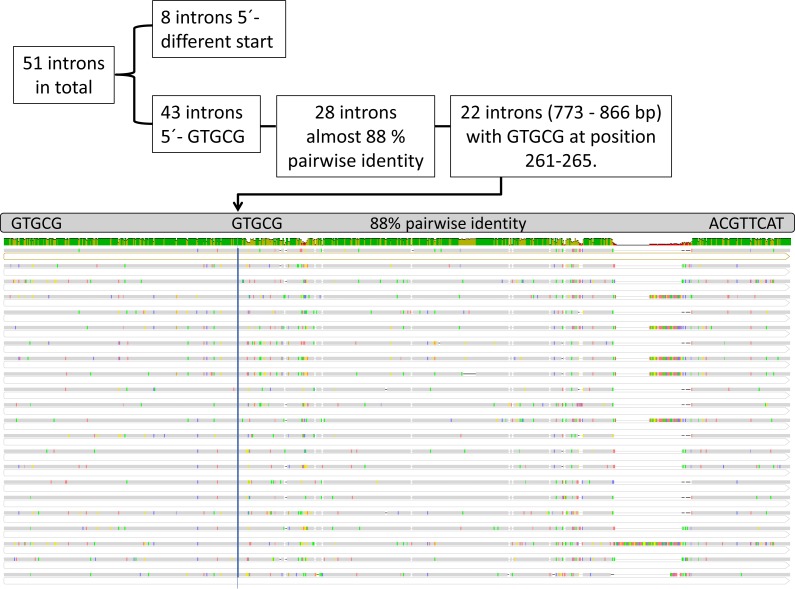
Alignment of group II introns. Intron identity according to boundaries and position of additional GTGCG (blue line). Geneious nucleotide alignment with absolute pairwise identities (green in first line, grey in alignment, different nucleotides in colored bars) of 21 introns in *Etl. pomquetensis* in various genes. Introns top down: *atp*B I2, *atp*B I3, *atp*B I4, *atp*E I2, *atp*H I1, *psa*A I1, *psa*A I2, *psa*A I3, *psa*A I4, *psa*A I6, *psa*B I2, *psa*C I3, *psb*B I2, *psb*C I3, *psb*C I5, *psb*D I1, *psb*D I5, *rbc*L I1, *rpl*32 I1, *rpo*B I1, *rps*7 I1, *rps*12 I1.

Twenty- two of these introns (773–866 bp) in *Etl. pomquetensis* additionally showed the same GTGCG motif at positions nt 261 to 265 upstream from base one of the intron, with pairwise identities of 88% (Fig. 3, Table S2 highlighted in gray).

We assume that all of these 28 introns of *Etl. pomquetensis* with high pairwise identity were closely related and arose from a single ancestor proliferating via retrotransposition and moved horizontally into DNA target sequences, which resembled the homing site. According to Lambowitz & Zimmerly (2011) and Lambowitz & Belfort (2015), retrotransposition to ectopic sites plays a major role in intron dissemination to novel locations, so that the many and very similar introns in *Etl. pomquetensis* could be explained.

### Possible proliferation of group II introns

Still the question remained of how these introns could be spliced without an ORF including maturase activity in domain IV. One possibility was that they rely on trans-acting RNAs or proteins with two feasible splicing mechanisms: (1) The introns of *Etl. pomquetensis* used host encoded proteins to promote splicing, reverse splicing and mobility, which is typical for most mitochondrial and plant chloroplast group II introns (Lambowitz & Zimmerly, 2011). *Chlamydomonas reinhardtii* even utilized nuclear-encoded maturases for splicing of the trans-spliced group II introns (Merendino et al., 2006).

(2) All these introns could be spliced by a single IEP (Intron-Encoded Protein) that could either be free-standing or located in a functional intron. This would provide an accessible splicing apparatus and allow all but one intron to lose its own IEP (Dai & Zimmerly, 2003; Lambowitz & Belfort, 2015; Lambowitz & Zimmerly, 2011). Brouard et al. (2016) assumed that the freestanding *orf1311* in *Oedocladium* (Chlorophyceae), with an intron encoded maturase, could function as promoter for splicing the ORF-less group II introns. Turmel, Otis & Lemieux (2016) detected introns in *G. planctonica* without ORFs, which may reflect an evolutionary pressure for a smaller and more compact intron structure enabling increased efficiency of splicing and mobility, when maturase activity is provided from elsewhere. Furthermore, it might be assumed that an early event in the *Etl. pomquetensis* cpGenome was the deletion of an intron encoded ORF, which appeared to have occurred prior to the spreading of introns across the genome and that other group II introns with encoded IEPs or freestanding ORFs acted *in trans* to promote splicing and mobility of ORF-free introns (Doetsch, Thompson & Hallick, 1998). To gain information about DNA target sites, which the introns of *Etl. pomquetensis* use for retrotransposition, we checked the insertion sites and the sequences of flanking exons. The exon nucleotides at the 5′-insertion site of the intron did not show any similarity, which might be due to a not strictly controlled transposition/ retrotransposition processes (Pombert et al., 2012), thus helping with random intron invasion all over the genome, and on both strands. The only conspicuous DNA target site the 28 homologous introns with high sequence similarity used for reverse splicing was a pyrimidine base, which represented the first nucleotide of the following exon (except for *atp*E exon 3, I2). The gene *psa*A contained the most of these introns, and five of six introns contained high similarity.

A search for related introns in *Etl. gymnastica, Et. viridis* and *P. parkeae* and all other euglenoid cpGenomes did not reveal any sequential or positional homology. Insertion sites found in *Etl. pomquetensis* were unique to that taxon.

The highest pairwise identity of introns was found in *E. gracilis var. bacillaris* with 56.7%, but only for three small 97 bp long group III introns (*rps*16 I1, *rpo*C1 I7 and *rps19* I2). Also, outside of euglenoid chloroplast introns, very few species showed high pairwise similarity. For instance, Brouard et al. (2016) found six group IIA introns in the chlorophyte *Oedocladium carolinianum* with high levels of nucleotide identities, which displayed over 80% pairwise identity. As well Turmel, Otis & Lemieux (2016) found several group II introns with high nucleotide identities also at various insertion sites, but only in small numbers. The introns of *ycf*3 and *psb*H in *Gloeotilopsis sarcinoides* were 85.6% identical. To our knowledge, *Etl. pomquetensis* is the first organism with more than 50 introns within protein coding genes and half of those sharing a pairwise identity of 90%.

### Lack of group III introns in the genome

The second peculiarity in *Etl. pomquetensis* is the absence of group III introns. Group III introns are believed to be the descendants of group II introns which only retained domains DI and DVI (Christopher & Hallick, 1989). The 5′ -boundaries are more variable than in group II introns, but most group III introns have a U at position 2 and a G at position 5. Most of them are of dyad symmetry near the 3′ -end similar to domain VI of group II introns. The motif driving the symmetry follows *abcdef* (3–8)*f*′*e*′*d*′*A*^∗^*c*′*b*′*a*′ (Drager & Hallick, 1993). The 3′ sequence of group II and III introns are variable, although the branch-point *A*^∗^ is usually at position eight, sometimes at seven, and occasionally at position nine. Interestingly, none of the 51 identified introns of *Etl. pomquetensis* complied with the typically confined group III intron size of 91–120 nucleotides (Christopher & Hallick, 1989; Copertino & Hallick, 1993; Doetsch, Thompson & Hallick, 1998; Drager & Hallick, 1993). Underpinning these findings, 43 of 51 introns started with a typical group II 5′-GTGCG (Table S2, start marked bold) and even the smallest intron was over 300 bp long (*rpo*C1 I1 356 bp). Furthermore, the intron size was even larger than group III twintrons (group III introns within group III introns), which were found in the chloroplast genome of *E. gracilis* (Copertino, Shigeoka & Hallick, 1992; Copertino et al., 1994). The smallest introns of *Etl. gymnastica* and *Et. viridis* were *rpo*B I1 with 179 bp (re-analyses of data from (Hrdá et al., 2012; Wiegert, Bennett & Triemer, 2012) and 156 bp, respectively, and these were larger than group III introns. Hence, we assumed that group III introns probably evolved after *Etl. pomquetensis* diverged. Secondary the structure of domain V and VI of *rpo*B I1 in *Etl. gymnastica* and *Et. viridis* was recognizable when some mismatches were allowed in the analyses (Fig. S4). Degeneration and mutation of group II introns in euglenoids have been described before and are known to impact secondary structure elements. Even domain V tolerates a surprising number of mismatches (Michel & Ferat, 1995). To our present knowledge, such introns best resemble mini-group II introns, which lack different domains (Doetsch, Thompson & Hallick, 1998). Under the presumption that *rpo*B I1 of *Etl. gymnastica* and *Et. viridis* are mini group II introns and not group III introns, we assume that group III introns evolved probably within the Euglenales after fresh-water and brackish environments became accessible together with warmer temperatures. The impact of the environmental medium could have been a driving force on degenerating group II introns. The change from group II intron to group III introns was observed in the *psb*C intron containing *mat*1 (*ycf*13). It is clearly a group II intron/ twintron in all Eutreptiales, but a group III twintron in *E. gracilis* with an open reading frame (*ycf*13, *mat*1) within the internal group III intron (Copertino et al., 1994; Table 4). Copertino et al. (1994) proposed that *mat*1 may be involved in group III intron metabolism and is required for group III intron excision and/or mobility in *Euglena* and *Astasia*. The ORF of *Euglena gracilis psb*C I4 has detectable similarity to the RT domain of group II intron ORFs, although it lacks characteristics of functional RT activity (Copertino et al., 1994; Doetsch, Thompson & Hallick, 1998; Mohr, Perlman & Lambowitz, 1993).

Based on the greater length of the *psb*C intron in *Etl. pomquetensis*, and a typical group II intron 5′ -boundary, it seems likely that the *psb*C intron is instead a group II intron/twintron. All three Eutreptiales have a *psbC* intron including *mat*1 (*ycf*13) that is at least three times larger than the group III twintron (I4) including *mat*1 of *E. gracilis* (Table 4). These findings, and the fact that *E. gracilis* contained a group II -type maturase in a group III twintron (Doetsch, Thompson & Hallick, 1998; Mohr, Perlman & Lambowitz, 1993), underpin the possibility that group II introns evolved first in basally branching euglenoid species. Subsequently, they degenerated by loss of different domains (in more derived species) to group III introns, containing only DI-like and DVI-like structures (Doetsch, Thompson & Hallick, 1998; Lambowitz & Belfort, 2015). This finding is also supported by identification of two maturase encoded introns and their predicted secondary structure models in *Lepocinclis buetschlii* by Doetsch, Thompson & Hallick (1998). The authors interpreted these introns as group II/group III intermediates just in the process of losing group II intron domains and they were designated as mini-group-II introns.

Summarizing, we presume that group II introns appeared first in an intron-less ancestral genome and gave rise to group III introns and from there on degeneration went on independently in different lineages. Further on, either the *Etl. pomquetensis* group II intron *mat*1 or another intron encoded protein (IEP) act *in trans* to promote splicing and mobility of ORF-less introns.

### Intron trends in Euglenoids

In their characterization of Euglenaceae, Bennett & Triemer (2015) noted that all Euglenaceae, but no Eutreptiales, contained an intron or twintron in *pet*B (I1) and that this intron/twintron may be a synapomorphy for at least the Euglenaceae. Kasiborski, Bennett & Linton (2016) identified a homologous intron/twintron within *pet*B I1 of *P. orbicularis* and discussed this intron/ twintron as a putative synapomorphy for the order Euglenales. However, in the cpGenome of *Etl. pomquetensis* two introns were detected in *pet*B. The first was found at the identical insertion site, but nearly two times larger than that of *E. gracilis* strain Z and five times larger than that of *P. orbicularis*. All *pet*B I1 introns started with a typical group II 5′ -GUGYG (*P. orbicularis* re-analysis, Table S1). This means, a group II intron in *pet*B could neither be a synapomorphy for the Euglenales, nor for the Euglenaceae, but evidently evolved at least in *Eutreptiella.*

### Twintron analysis

**Figure 4:**
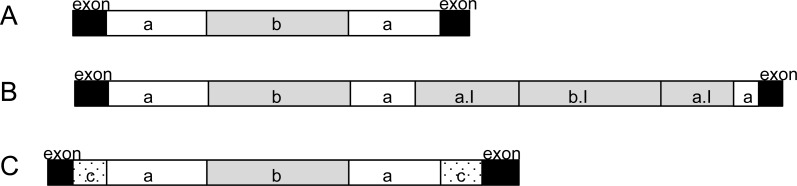
Analysis of potential twintrons with high sequence similarity. (A) Highly conserved introns are shown. (B) Structure of the *pet*G I1 complex twintron. (C) Structure of *psa*C I2. Black boxes represent exons. White boxes (a) are external introns of twintrons, white dotted boxes (c) are external introns of complex twintrons. Grey boxes (b, a.I, b.I) represent internal introns, whereby a.I showed high sequence similarity to external intron a and b.I to internal intron b.

All 51 external introns were investigated for the presence of potential twintrons using a Python script, which searched for the conserved 3′ motif of group II and group III introns reported in Copertino & Hallick (1993). The search resulted in 28 external introns which contained at least one 3′ motif (see GenBank accession). Sixteen of the 28 introns contained four kinds of repeated 3′ motifs (Table S2, indicated by number of asterisks). Additionally, four potential group II twintrons were found (*rpo*B I1, *rps*2 I2, *psb*C I2, *psb*D I4, added to annotation) with only one 3′ motif and only one 5′ -GUGYG prior to the identified 3′ motif. Two of these potential group II twintrons (*rpo*B I1 and *psb*D I4) were those which share strong nucleotide identity with half of the introns detected in *Etl. pomquetensis*. We assume that all 28 introns (Table S2 marked bold except for *pet*G I1 and *psa*C I2) with equal intron organization (5′ motif GTGCG, 3′ motif ACGTTCAT and further GTGCG at nt 261-265) are potential twintrons with an external and internal group II intron (Fig. 4A). Secondary structure analysis of domain V and VI of the potential internal introns of *rpo*B I1and *psb*D I4 in *Etl. pomquetensis* showed recognizable counterparts, when mismatches were allowed in the analyses (Fig. S5). For the potential internal introns in *rpo*B I1 and *psb*D I4 the conserved three base pairs (5′- …AGC …−3′) near the base of the stem of domain V were detectable, but the secondary structure showed a slightly altered terminal loop and no branch-point *A*^∗^ was detectable in domain VI (Michel & Ferat, 1995; Thompson et al., 1997). Since the Phyton script only detects an unaltered conserved 3′ motif, only two of the close related introns have been detected as potential twintrons. This underpins several statements, that group II introns of phototrophic euglenoids are highly degenerated and persistent to detailed analysis (Michel & Ferat, 1995; Mohr, Ghanem & Lambowitz, 2010). Two introns, *psa*C I2 and *petG* I1, out of the 28 potential twintrons with high sequence similarity were significantly larger and thus investigated for the presence of potential complex twintrons.

*psa*C I2 analysis: The intron *psa*C I2 of *Etl. pomquetensis* was 1,294 bp long and by this more than 400 bp longer than the average. The nucleotide sequence alignment of all 28 introns (Table S2) was remarkably well conserved. It showed that *psa*C I2 is a complex twintron with an external intron interrupted by the same potential internal twintron as all the others (Figs. 4A and 4C).

The potential internal twintron (825 bp) shared 88% pairwise identity with the other 27 potential twintrons (Table S2). It is located 281 bp downstream of the external 5′ splice site. Comparing the secondary structure of domain V of the external intron a of the internal twintron (Fig. 4C) with the other highly conserved twintrons (Fig. 4A) resulted in identical stems and loops with only two out of 34 nucleotides differing (Fig. S3).

A BLASTn search for the external intron of *psa*C I2 (Fig. 4C dotted intron c) revealed weak similarity with *psb*C I2 (containing a still unspecified maturase) of *Etl. gymnastica*. Secondary structure analysis of domains V and VI of *psb*C I2 from *Etl. gymnastica*, realigned by Dabbagh & Preisfeld (2016), and the external intron of *psa*C I2 in *Etl. pomquetensis* revealed highly conserved structures of domains V (Fig. S6). They only differed in six nucleotides and contained the AGC motif near the base of the stem from the 5′-boundary (Thompson et al., 1997). We presume that the external intron of *psa*C I2 in *Etl. pomquetensis* (Fig. 4C dotted intron c) is closely related to and arose from the same ancestral intron as *psb*C I2 in *Etl.gymnastica* and that the intron degeneration and loss of the maturase in *Etl. pomquetensis* took place afterwards.

*pet*G I1 analysis: We were also interested in closely investigating *pet*G I1, because it was more than twice the size of all other highly conserved potential twintrons, but shared pairwise identities of 87.4%. This resulted in the identification of *pet*G I1 as a complex twintron with high pairwise identities of internal and external twintrons (Fig. 4B). The two twintrons in *pet*G I1 were the same and showed 90% pairwise identity. Both started with a 5′-GTGCG boundary, a 3′ -boundary ACGTTCAT motif and an additional GTGCG at insertion site 261. A comparison of the secondary structure of the introns (Fig. 4B intron a/ intron a.I) with the consensus domain V from the other highly conserved potential twintrons (Fig. S3) showed that 33 out of the 34 nucleotides were identical. The internal twintron comprised 799 bp and was located three nucleotides upstream from the 3′ splice site of the external twintron. It seems reasonable that the internal twintron proliferated into the external twintron and that both originated from the same twintron as the other ones (Figs. 4A and 4B).

## Conclusion

Analysis of the genome of all euglenoids sequenced so far in regard to sequence and structural levels makes it apparent that the green algae origin is most visible in the cpGenome of *Etl. pomquetensis*. This can be seen by high pairwise identities in coding regions with the putative chloroplast ancestor *P. parkeae* and a typical green algae and land plant quadripartite genome structure. Still, independent evolution of the genomes since secondary endosymbiosis can also be observed in *Etl. pomquetensis* by decreased protein coding gene content and increased intron numbers compared to *P. parkeae*.

The cpGenome size of *Etl. pomquetensis* was substantially larger than those of other Eutreptiales published so far due to an increased number of introns and intergenic space, and was closest in size to the largest known euglenoid cpGenomes. This contradicts earlier assumptions that introns invaded cpGenomes massively in Euglenales. Interestingly, and unique within the phototrophic euglenoids, we detected a high similarity between more than half of the 51 introns. Another singularity was that no group III introns, or group III twintrons could be identified. This underlines the hypothesis that group II introns arrived first in basally branching euglenoid species and group III introns emerged from group II introns.

Finally, we speculate that future investigations could explore the possibility of a psychrophilic member of the *Pyramimonas* genus as a putative chloroplast donor to the euglenoid lineage and that *Etl. pomquetensis* may very well be the nearest relative up to date.

##  Supplemental Information

10.7717/peerj.3725/supp-1 Figure S1 Progressive Mauve analysis of Eutreptiales Each box represents a cluster of homologous genes with *Eutreptiella pomquetensis* as the reference genome. Like blocks are labelled by letters A-J. See Table 4 for a list of genes contained in each block. In the Mauve alignment the repeat regions of rRNA were not included, because Mauve will not align repeat regions, which have multiple matches on both genomes.

10.7717/peerj.3725/supp-2 Figure S2 Progressive Mauve analysis comparing the cpGenomes of *Etl. pomquetensis* and three Chlorophyta Each box represents a cluster of homologous genes between *Eutreptiella pomquetensis* as the reference genome** and* Pyramimonas parkeae* (A), *Pycnococcus provasolii* (B) and *Ostreococcus tauri* (C). In the Mauve alignment the repeat regions of rRNA were not included, because Mauve will not align repeat regions, which have multiple matches on both genomes.

10.7717/peerj.3725/supp-3 Figure S3 Consensus secondary structure model of domain V and VI of highly conserved introns Consensus secondary structure model of domain V and VI of the highly conserved introns of *Etl. pomquetensis* based on the model proposed by Michel, Umesono & Ozeki, (1989) and on comparative analysis of other euglenoid group II introns (Thompson et al., 1997). The three base pairs (5’- ...AGC ... GUU…-3’) near the base of stem V were invariant (red box). Introns that form consensus sequence: atpB I1- I4; *atp* E I2; *atp* H I1; *psa* A I1- I4 & I6; *psa* B I1-I2; *psa* C I3; *psb* B I2; *psb* C I3 & I5; *psb* D I1& I4-I5; *rbc* L I1; *rpl* 32 I1; *rpo* B I1& I3; *rps* 7 I1; *rps* 12 I1.

10.7717/peerj.3725/supp-4 Figure S4 Putative secondary structure model of domain V and VI of supposed mini-group II intron Domain V and VI of *rpo* B I1 of *Et. viridis* (A) with branch-point A* at position 8 of domain VI and conserved three base pairs (5’- …AGC …-3’) near the base of the stem of domain V (red box). Domain V and VI of *r po* B I1 of *Etl. gymnastica* (B) with slightly altered base pairs (5’- …AGUC …-3’) of domain V (red box) but without branch-point A* at position 8 of domain VI.

10.7717/peerj.3725/supp-5 Figure S5 Secondary structure model of potential internal introns of twintrons Internal introns of *rpo* B I1 of *Etl. pomquetensis* (A) with conserved three base pairs (5’- …AGC …-3’) near the base of the stem of domain V (red box). Internal intron of *psb* D I4 of *Etl. pomquetensis* (B) with conserved three base pairs (5’- …AGC …-3’) near the base of the stem of domain V (red box).

10.7717/peerj.3725/supp-6 Figure S6 Comparative secondary structure analysis Secondary structure model of putative domain V and VI of *psa* C I2 external intron of *Etl. pomquetensis* (A) with branch-point A* at position 7 of domain VI and conserved three base pairs (5’- …AGC …-3’) near the base of the stem of domain V (red box). Secondary structure model of putative domain V and VI of *psb* C I2 of *Etl. gymnastica* (B) with slightly altered branch-point AU* at positions 7 and 8 of domain VI and conserved three base pairs (5’- …AGC …-3’) near the base of the stem of domain V (red box).

10.7717/peerj.3725/supp-7 Table S1 CpGenome features of euglenoids and depicted prasinophytes according to NCBI annotation * Chloroplast circle not closed. a Twintrons were counted as single insertion sites. b Including rRNA repeats and intermediate tRNAs. c Includes the identified 5S, for S. acuminata the two identified 5S. d Includes the two introns in rps18. e Includes the one intercistronic intron rps4-rps11. f First exon could not be identified, so gene length is a minimum. g Realigned, but with alternative start codon. h Start codon not determined, due to undetermined exon1 i: New intron start after re-analyses

10.7717/peerj.3725/supp-8 Table S2 Features of introns in protein-coding genes of Etl. pomquetensis

10.7717/peerj.3725/supp-9 Supplemental Information 1 DNA Sequence for review

10.7717/peerj.3725/supp-10 Supplemental Information 2 GenBank annotation preview for review, raw data

## Additional Information and Declarations

### DNA Deposition

The following information was supplied regarding the deposition of DNA sequences: The sequences have been uploaded as Supplemental Files. The data is also available under GenBank accession number KY706202.

### Data Availability

The following information was supplied regarding data availability: The raw data has been supplied as a Supplementary File.
