# Supplementary figures and images for: Chloroplast genome expansion by intron multiplication in the basal psychrophilic euglenoid Eutreptiella pomquetensis
Source: PeerJ. 2017 Aug 25;5:e3725. doi: 10.7717/peerj.3725 (PMC5572947; doi:10.7717/peerj.3725)

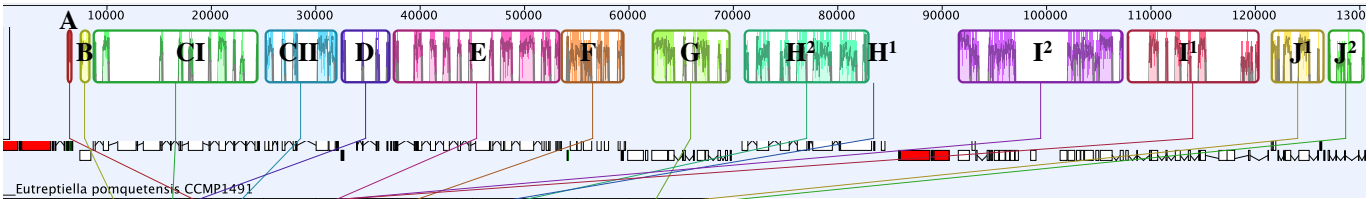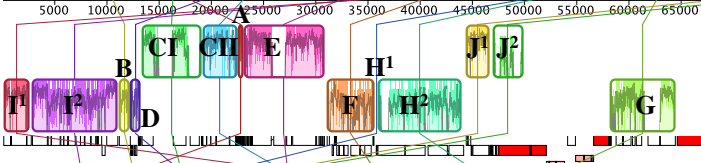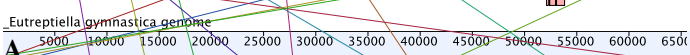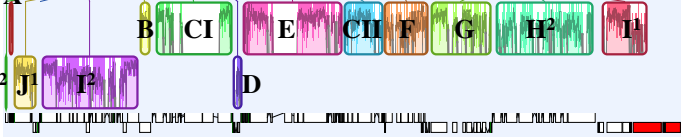

Supplement: Figure S1 — Each box represents a cluster of homologous genes with Eutreptiella pomquetensis as the reference genome. Like blocks are labelled by letters A-J. See Table 4 for a list of genes contained in each block. In the Mauve alignment the repeat regions of rRNA were not included, because Mauve will not align repeat regions, which have multiple matches on both genomes. [file peerj-05-3725-s001.pdf]

A

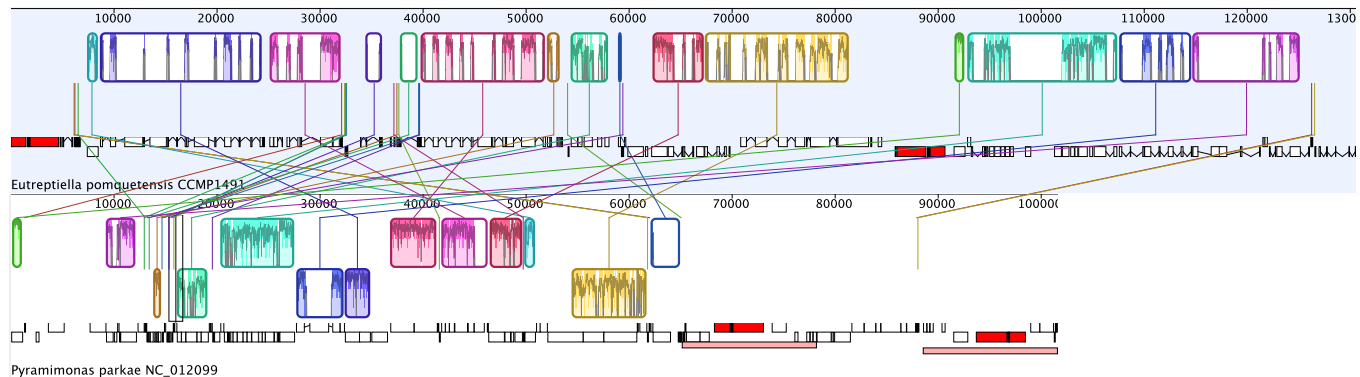

B

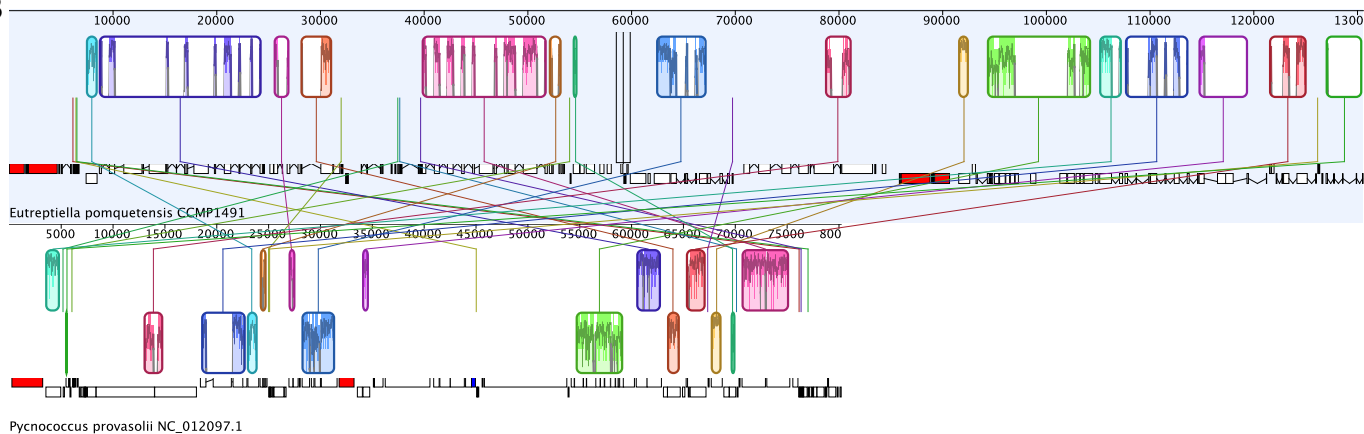

C

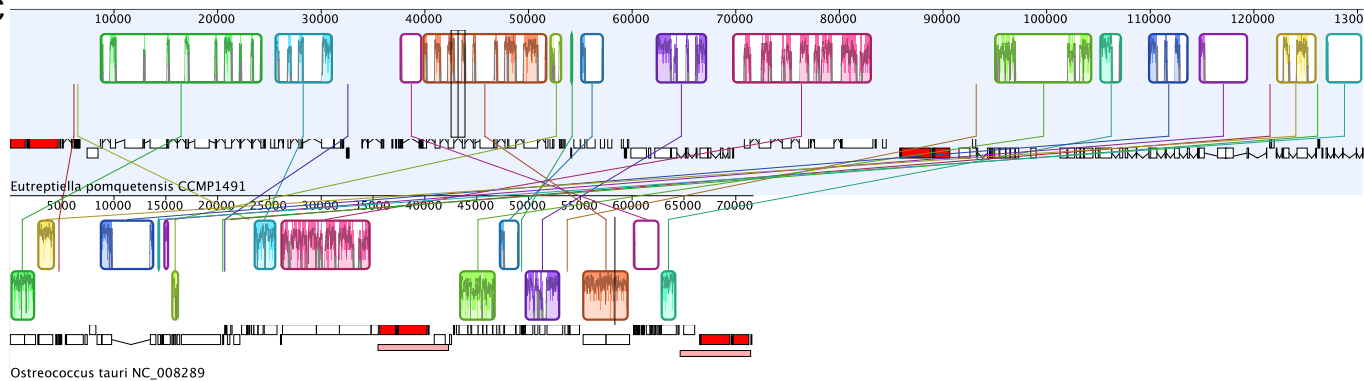

Supplement: Figure S2 — Each box represents a cluster of homologous genes between Eutreptiella pomquetensis as the reference genome and Pyramimonas parkeae (A), Pycnococcus provasolii (B) and Ostreococcus tauri (C). In the Mauve alignment the repeat regions of rRNA were not included, because Mauve will not align repeat regions, which have multiple matches on both genomes. [file peerj-05-3725-s002.pdf]

domain VI

domain  $V$

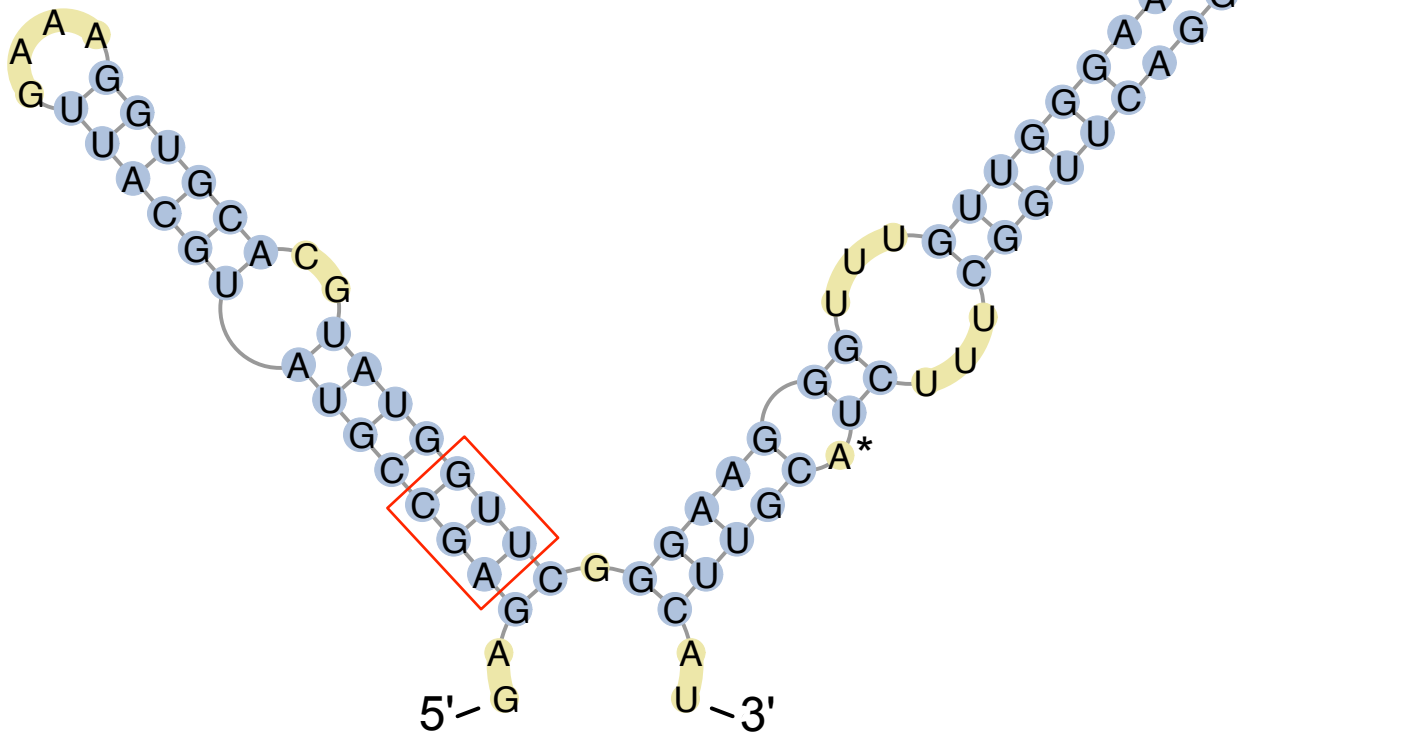

Supplement: Figure S3 — Consensus secondary structure model of domain V and VI of the highly conserved introns of Etl. pomquetensis based on the model proposed by Michel, Umesono & Ozeki, (1989) and on comparative analysis of other euglenoid group II introns (Thompson et al., 1997). The three base pairs (5’- ...AGC ... GUU…-3’) near the base of stem V were invariant (red box). Introns that form consensus sequence: atpB I1- I4; atp E I2; atp H I1; psa A I1- I4 & I6; psa B I1-I2; psa C I3; psb B I2; psb C I3 & I5; psb D I1& I4-I5; rbc L I1; rpl 32 I1; rpo B I1& I3; rps 7 I1; rps 12 I1. [file peerj-05-3725-s003.pdf]

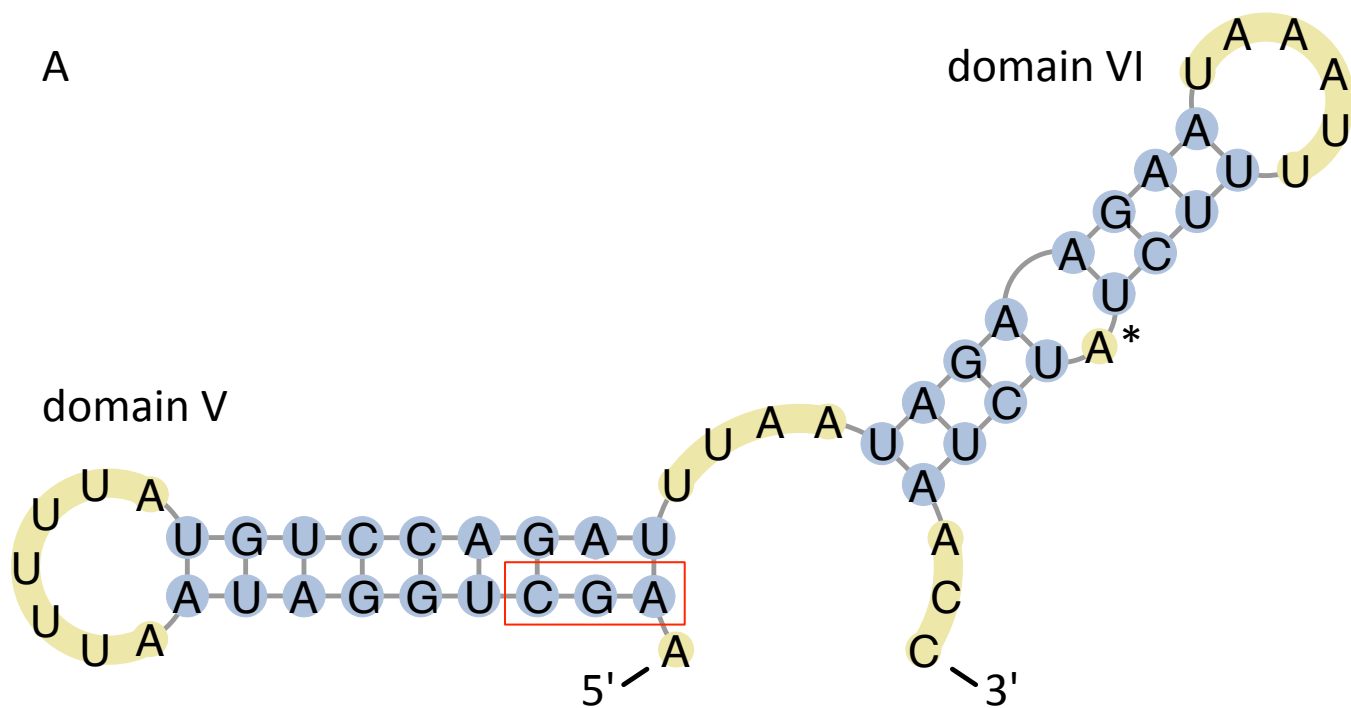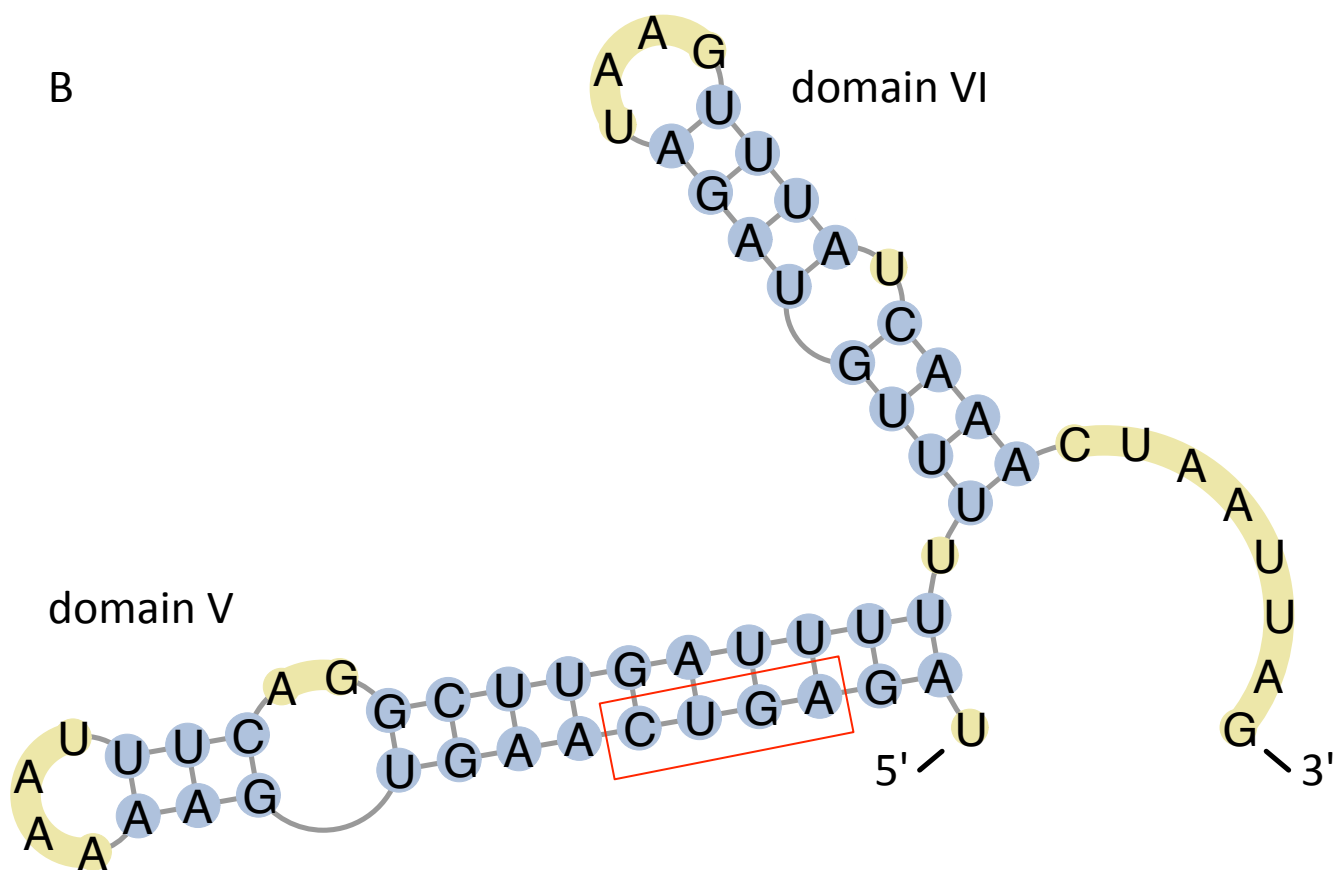

Supplement: Figure S4 — Domain V and VI of rpo B I1 of Et. viridis (A) with branch-point A* at position 8 of domain VI and conserved three base pairs (5’- …AGC …-3’) near the base of the stem of domain V (red box). Domain V and VI of r po B I1 of Etl. gymnastica (B) with slightly altered base pairs (5’- …AGUC …-3’) of domain V (red box) but without branch-point A* at position 8 of domain VI. [file peerj-05-3725-s004.pdf]

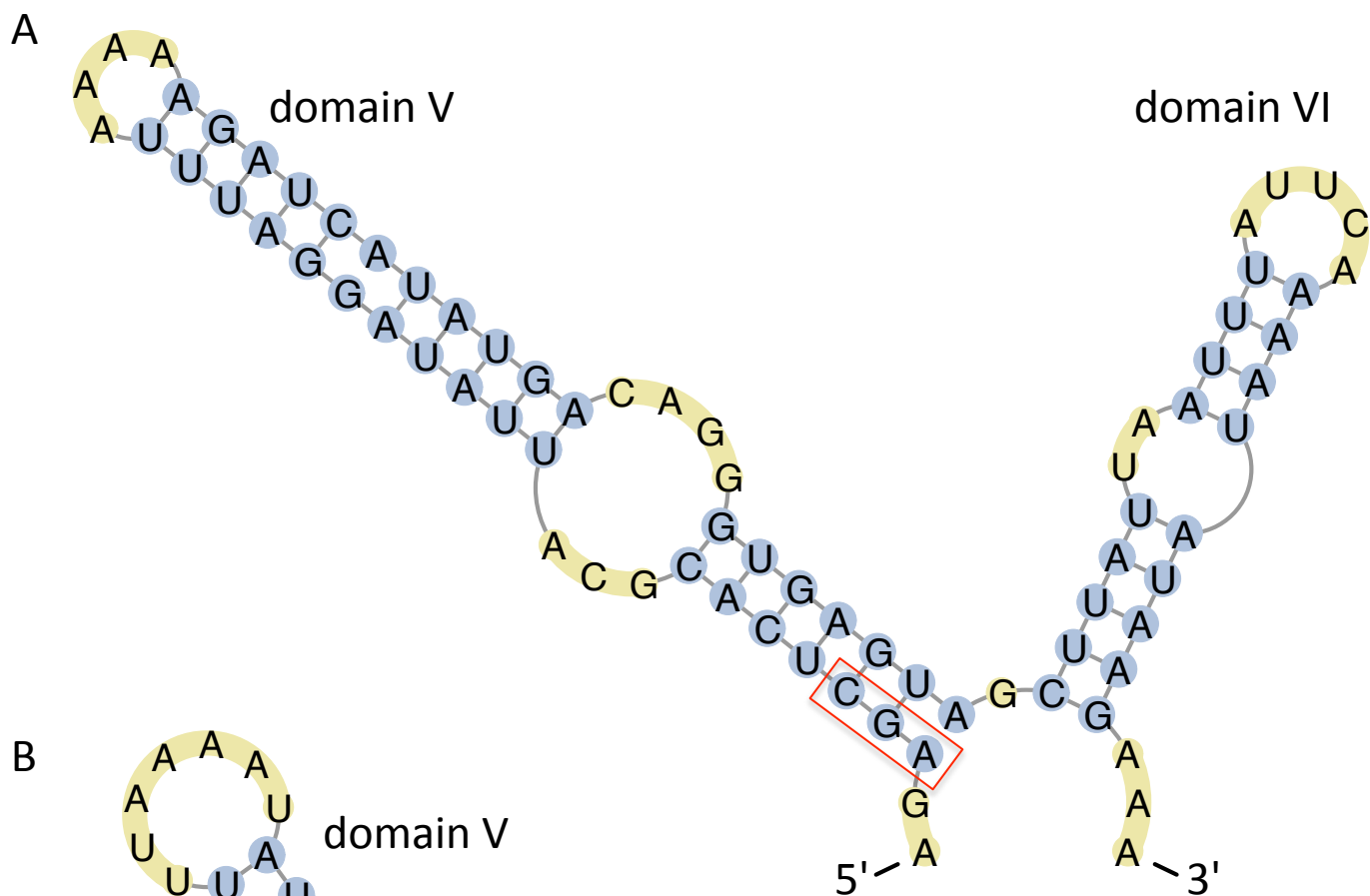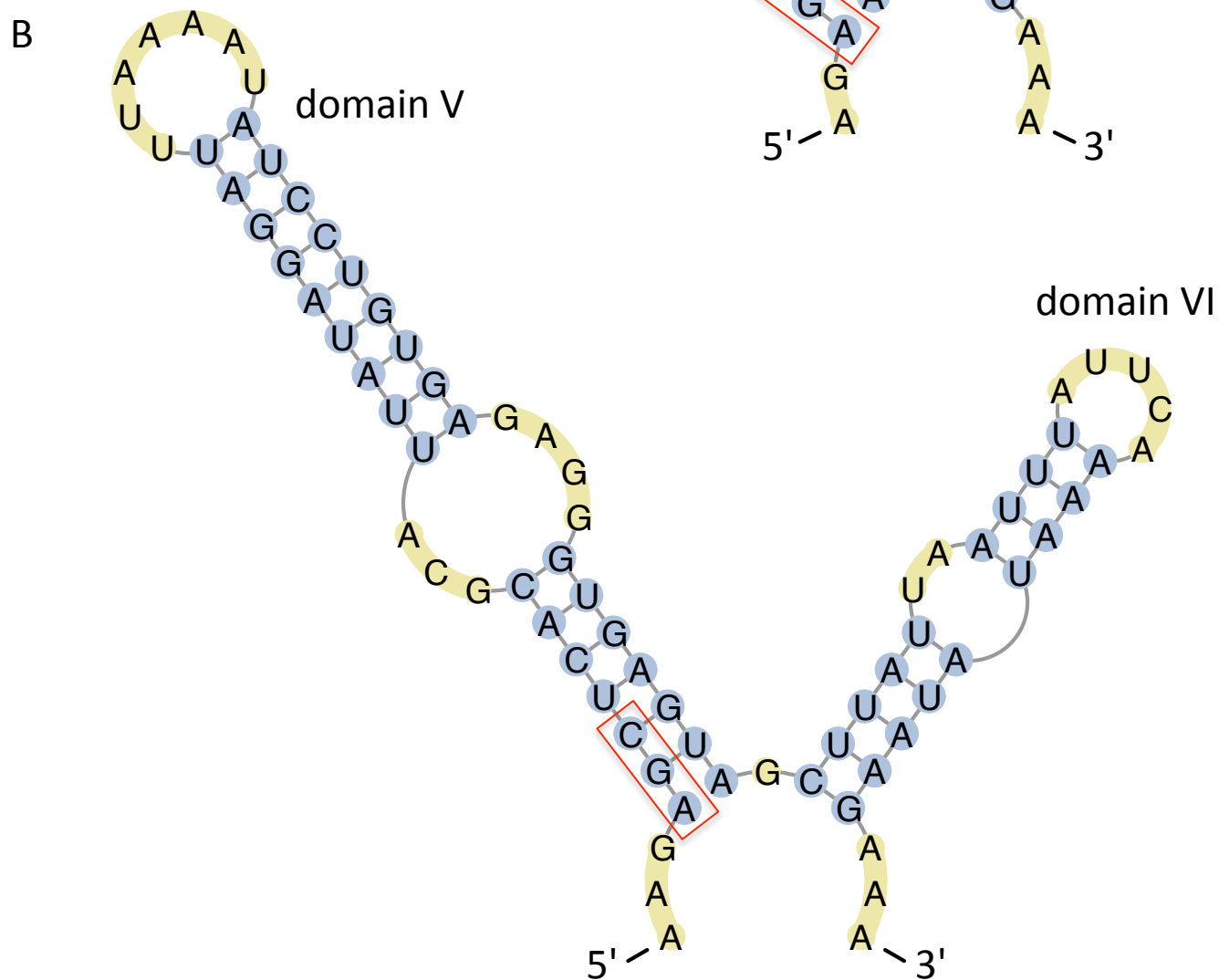

Supplement: Figure S5 — Internal introns of rpo B I1 of Etl. pomquetensis (A) with conserved three base pairs (5’- …AGC …-3’) near the base of the stem of domain V (red box). Internal intron of psb D I4 of Etl. pomquetensis (B) with conserved three base pairs (5’- …AGC …-3’) near the base of the stem of domain V (red box). [file peerj-05-3725-s005.pdf]

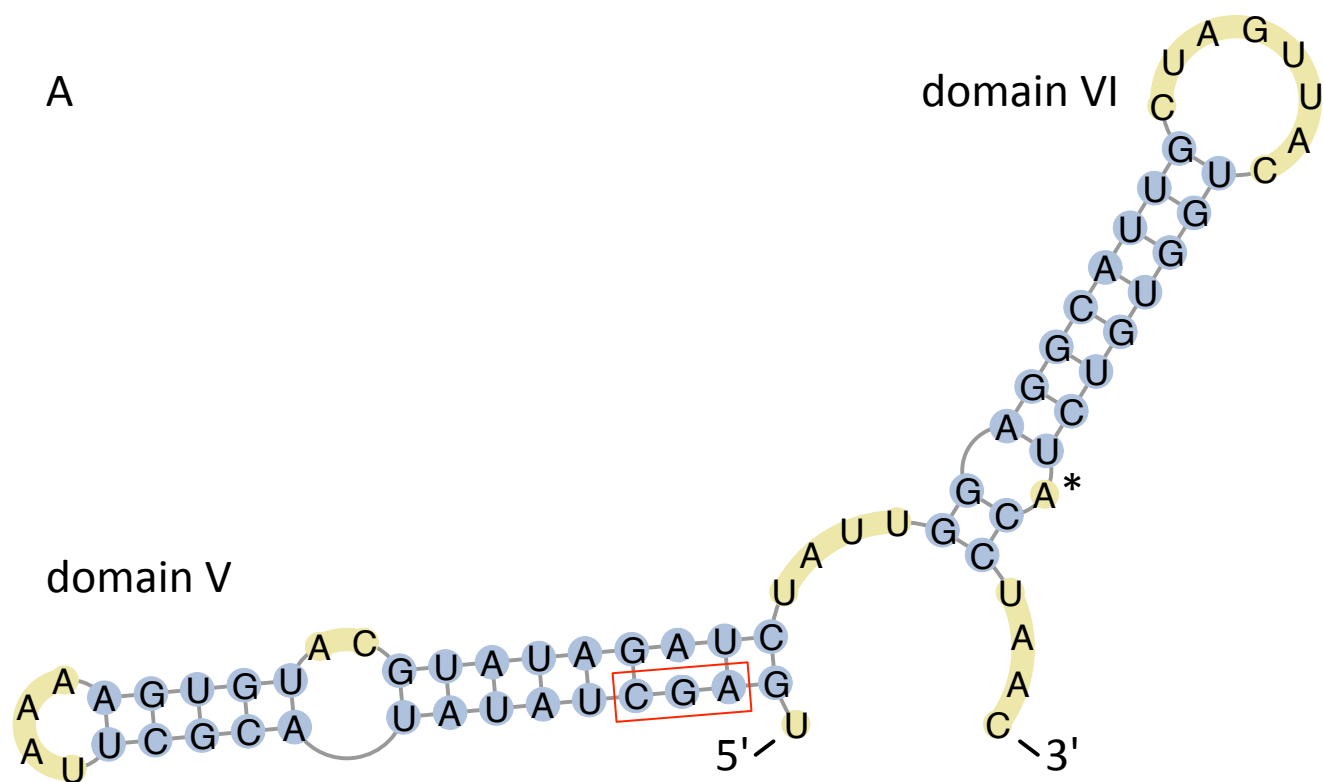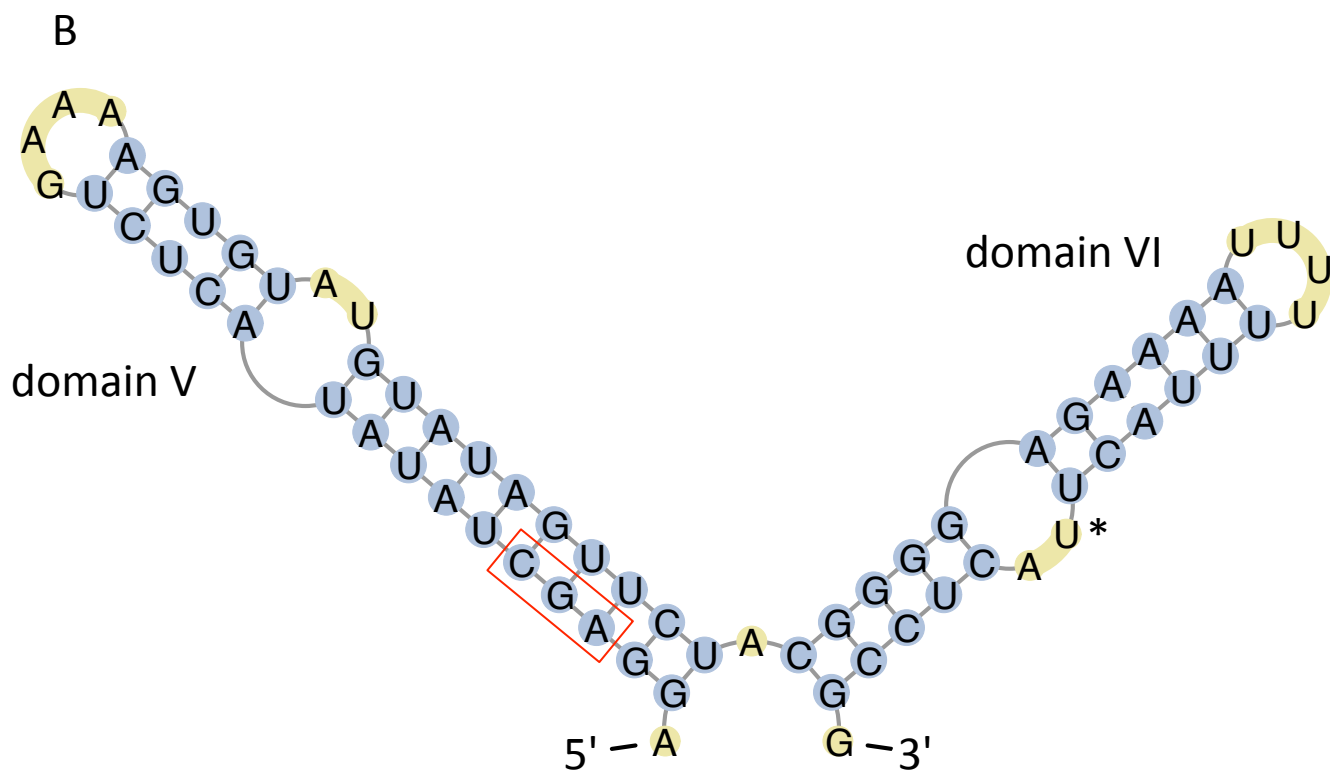

Supplement: Figure S6 — Secondary structure model of putative domain V and VI of psa C I2 external intron of Etl. pomquetensis (A) with branch-point A* at position 7 of domain VI and conserved three base pairs (5’- …AGC …-3’) near the base of the stem of domain V (red box). Secondary structure model of putative domain V and VI of psb C I2 of Etl. gymnastica (B) with slightly altered branch-point AU* at positions 7 and 8 of domain VI and conserved three base pairs (5’- …AGC …-3’) near the base of the stem of domain V (red box). [file peerj-05-3725-s006.pdf]
