# Supplementary material for: Chloroplast genome expansion by intron multiplication in the basal psychrophilic euglenoid Eutreptiella pomquetensis
Source: PeerJ. 2017 Aug 25;5:e3725. doi: 10.7717/peerj.3725 (PMC5572947; doi:10.7717/peerj.3725)
Supplement: Table S1 — * Chloroplast circle not closed. a Twintrons were counted as single insertion sites. b Including rRNA repeats and intermediate tRNAs. c Includes the identified 5S, for S. acuminata the two identified 5S. d Includes the two introns in rps18. e Includes the one intercistronic intron rps4-rps11. f First exon could not be identified, so gene length is a minimum. g Realigned, but with alternative start codon. h Start codon not determined, due to undetermined exon1 i: New intron start after re-analyses [file peerj-05-3725-s007.docx]

| Taxon | Size (bp) | A+T % | Genes | Introns^a^ | ORFs | roaA | CDS of rpoA (bp) | Largest gene (bp) | Shortest gene (bp) | Gene with most introns | psbD/psbC overlap | petB I1  bp/5´start |
| --- | --- | --- | --- | --- | --- | --- | --- | --- | --- | --- | --- | --- |
| *E. gracilis* strain *Z* | 143,171 | 73.9 | 116^b^ | 134 | 7 | + | 651 | *psb*C (10861) | psaM (96) | rpoC1 (11) | + | 909/GUGCG |
| *E. gracilis* var*. bacillaris* | 132,034* | 74.2 | 104 | 134 | 6 | + | 525 | psbC (11446) | psaM (96) | rpoC1 (11) | + | 909/GUGCG |
| *E. viridis* | 76,156 | 73.8 | 92 | 77 | 0 | + | 483 | psbC (6165) | *psa*M (96) | rpoB (10) | + | 528/GUGUG |
| *E. viridis epitype* | 91,616 | 73.6 | 92 | 76 | 13 | + | 480 | psbC (6131) | psaM (96) | rpoC1  (8) | + | 533/GUGUG |
| *E. mutabilis* | 86,975 | 73.3 | 91 | 77^e^ | 0 | + | 636 | psbC (12192) | psaM (96) | rpoC1 (9) | + | 435/GUGCG |
| *Era. anabaena* | 88,487 | 72 | 93 | 82 | 4 | + | 624 | psbA (6998) | psaM (96) | rpoB (10) | + | 551/GUGCG |
| *M. parapyrum* | 80,147 | 72 | 93 | 80 | 1 | + | 507 | psbC (6127) | psaM (96) | rpoB/-C1 (9) | + | 441/GUGCG |
| *M. aenigmatica* | 74,746 | 70.6 | 93^c^ | 53 | 1 | + | - | psbC (6229) | psaM (96) | rpoC1 (9) | + | 544/GUGCG |
| *Cr. skujae* | 106,843 | 73.7 | 93 | 84 | 4 | + | 489 | psbC (8947) | psaM (96) | rpoB (10) | + | 537/GUGUG |
| *S. acuminata* | 144,166* | 73.4 | 95^c^ | 112^d^ | 0 | + | 486 | psaB (11283) | psbT (90) | rpoB/ rbcL (9) | + | 510/GUGCG |
| *T. volvocina* | 85,392* | 72.7 | 93 | 94 | 1 | + | 543 | psbC (7698) | psaM (96) | rpoC1 (11) | + | 536/GUGCG |
| *C. vesiculosum* | 128,892* | 73.9 | 92^c^ | 128 | 6 | + | - | psbC (11567) | psbI (105) | rpoC1 (11) | + | 470/GUGUG |
| *Efs. proxima* | 94,185* | 73.1 | 91 | 113 | 2 | + | - | psbC^f^(6648) | psaM (96) | rpoB (10) | ^h^ | 478/GUGCG |
| *P. orbicularis* | 65,992 | 72,8 | 91 | 66 | 1 | + | - | rpoB (3993) | psaM (96) | rpoB (10) | ^+^ | 357/GUGCG^i^ |
| *Et. viridis* | 65,523* | 71.4 | 83 | 24 | 3 | - | 672 | psbC (5706) | psbT (96) | rpoB (5) | - | - |
| *Etl. gymnastica* | 67,623 | 65.7 | 89 | 7 | 4 | - | 846 | psbC | psaM/psbT (96) | psbC (2) | +^g^ | - |
| ***Etl. pomquetensis*** | **130,561*** | **64.9** | **94** | **51** | **10** |  |  | **psbD (8412)** | **psaM (96)** | **psaA (6)** | **+** | **1736/GUGUG** |
| *Pyramomonas parkae* | 101,605 | 65.3 | 125 | 1 | 5 | - | 1056 | atpB (4224) | psbT (96) | atpB (1) | + | - |
| *Pyconococcus provasolii* | 80,211 | 60.5 | 98 | 1 | 3 | - | 1074 | ftsH (7944) | psbT (96) | atpB (1) | + | - |
| *Ostreococcus tauri* | 71,666 | 60.1 | 92 | 1 | 2 | - | 1071 | atpB (5188) | psaM/psbT (96) | atpB (1) | + | - |

* Chloroplast circle not closed.

a Twintrons were counted as single insertion sites.

b Including rRNA repeats and intermediate tRNAs.

c Includes the identified 5S, for S. acuminata the two identified 5S.

d Includes the two introns in rps18.

e Includes the one intercistronic intron rps4-rps11.

f First exon could not be identified so gene length is a minimum.

g Realigned, but with alternative start codon.

h Start codon not determined, due to undetermined exon1

i: new intron start after re-analyses
