# Supplementary material for: Chloroplast genome expansion by intron multiplication in the basal psychrophilic euglenoid Eutreptiella pomquetensis
Source: PeerJ. 2017 Aug 25;5:e3725. doi: 10.7717/peerj.3725 (PMC5572947; doi:10.7717/peerj.3725)
Supplement: Supplemental Information 2 [file peerj-05-3725-s010.docx]

GenBank Preview KY706202

Eutreptiella_pomquetensis130561 bp DNA linear

DEFINITION Eutreptiella pomquetensis.

ACCESSION

VERSION

KEYWORDS .

SOURCE plastid Eutreptiella pomquetensis

ORGANISM Eutreptiella pomquetensis

Unclassified.

REFERENCE 1 (bases 1 to 130561)

AUTHORS Dabbagh,N., Bennett,M.S., Triemer,R.E. and Preisfeld,A.

TITLE Chloroplast genome expansion by intron multiplication in the basal

psychrophilic euglenoid Eutreptiella pomquetensis

JOURNAL Unpublished

REFERENCE 2 (bases 1 to 130561)

AUTHORS Dabbagh,N., Bennett,M.S., Triemer,R.E. and Preisfeld,A.

TITLE Direct Submission

JOURNAL Fachbereich 4, Zoology and Didactics of

Biology, Bergische University Wuppertal, Gaussstrasse 20, Wuppertal

42119, Germany

FEATURES Location/Qualifiers

source 1..130561

/organism="Eutreptiella pomquetensis"

/organelle="plastid"

/mol_type="genomic DNA"

gene 1..1484

/gene="rrn16"

rRNA 1..1484

/gene="rrn16"

/product="16S ribosomal RNA"

gene 1576..1649

/gene="trnI"

tRNA 1576..1649

/gene="trnI"

/product="tRNA-Ile"

gene 1709..1781

/gene="trnA"

tRNA 1709..1781

/gene="trnA"

/product="tRNA-Ala"

/anticodon=(pos:1742..1744,aa:Ala,seq:tgc)

gene 1836..4706

/gene="rrn23"

rRNA 1836..4706

/gene="rrn23"

/product="23S ribosomal RNA"

gene 4767..4882

/gene="rrn5"

rRNA 4767..4882

/gene="rrn5"

/product="5S ribosomal RNA"

gene 5016..5977

/gene="rpl32"

CDS join(5016..5107,5899..5977)

/gene="rpl32"

/codon_start=1

/transl_table=11

/product="ribosomal protein L32"

/translation="MAVPKKKMSKSKKNMRKSVWKQKASKQATLALSLAKSVLSGNSK

GFLYLSSDSVEN"

exon 5016..5107

/gene="rpl32"

/number=1

intron 5108..5898

/gene="rpl32"

/number=1

misc_feature 5242..5266

/gene="rpl32"

/note="3' motif for potential twintron"

misc_feature 5332..5354

/gene="rpl32"

/note="3' motif for potential twintron"

misc_feature 5639..5658

/gene="rpl32"

/note="3' motif for potential twintron"

exon 5899..5977

/gene="rpl32"

/number=2

gene 6138..6209

/gene="trnH"

tRNA 6138..6209

/gene="trnH"

/product="tRNA-His"

/anticodon=(pos:6170..6172,aa:His,seq:gtg)

gene 6276..6347

/gene="trnM"

tRNA 6276..6347

/gene="trnM"

/product="tRNA-Met"

/anticodon=(pos:6308..6310,aa:Met,seq:cat)

gene 6398..6470

/gene="trnW"

tRNA 6398..6470

/gene="trnW"

/product="tRNA-Trp"

/anticodon=(pos:6431..6433,aa:Trp,seq:cca)

gene 6538..6610

/gene="trnE"

tRNA 6538..6610

/gene="trnE"

/product="tRNA-Glu"

/anticodon=(pos:6572..6574,aa:Glu,seq:ttc)

gene 6628..6700

/gene="trnG"

tRNA 6628..6700

/gene="trnG"

/product="tRNA-Gly"

/anticodon=(pos:6661..6663,aa:Gly,seq:tcc)

gene complement(7403..8458)

/gene="chlI"

CDS complement(7403..8458)

/gene="chlI"

/codon_start=1

/transl_table=11

/product="chlorophyll biosynthesis"

/translation="MVEQTNERPVFPFTSIVGQEEMKLALILNVIDPKIGGVMIMGDR

GTGKSTTVRALVDLLPQIEVATNDQFNSDPYDTELMSDAIRNMVRENKEIPVSKIKIP

MVDLPLGATEDRVCGTIDIEKALTEGVKAFEPGLLAKANRGILYVDEVNLLDDHLVDV

LLDSAASGWNTVEREGISICHPARFILVGSGNPEEGELRPQLLDRFGMHAQIGTVKEP

DLRVKIVEQRALFDQNPHEFRAEYKNEQEALMSQIVEARERLIRVKIDYDLRLKISEI

CSELNVDGLRGDMVTNRAAKALVAFEGRDEVTPRDIYTIITLCLRHRLRKDPLESIDS

GSKVQDVFNKAFGFDEE"

CDS join(8682..8873,9652..10150,12895..12929,15165..15285,

16104..16126,16905..17093)

/codon_start=1

/transl_table=11

/product="photosystem II core 34 kDa protein"

/translation="MTIAIGKTEEKRGLFDAADDWLKRDRFVFVGWSGLLLFPCAYLA

LGGWLTGITFVTSWYTHGLASSFLEGCNVLTAAVSTPPNSMAHSLLLLWGPEAQGEFT

RWLQLGGLWPFVALHGAFALIGFMLRQFEIARAVQIRPYNAIAFSAPIAVFVSVFLIY

PLGQSGWFFAPSFGVASIFRFILFFQGFHNWTLNPFHMMGVAGVLGAALLCAIHGATV

ENTLFEDGDGSNTFRAFNPTQAEETYSMVTANRFWSQIFGVAFSNKRWLHFFMLFVPV

TGLWMSAIGVVGLALNLRAYDFVSQEIRAAEDPEFETFYTKNILLNEGIRAWMAAQDQ

PHEQLVFPEEVLPRGNAL"

exon 8682..8873

intron 8874..9651

/gene="psbD"

/number=1

misc_feature 9098..9120

/note="3' motif for potential twintron"

exon 9652..10150

/gene="psbD"

/number=2

intron 10151..12894

/gene="psbD"

/number=2

misc_feature 10942..10961

/note="3' motif for potential twintron"

CDS 11034..12788

/note="group II intron reverse transcriptase/maturase"

/codon_start=1

/transl_table=11

/product="putative reverse transcriptase and intron

maturase"

/translation="MSIKTWLDVDWALCEQRVYRLQKRIFTASQAENKGKVHFLQRKI

LCSLDAKLIAVRRITQINKGRKIPGIDKKRIKNNKEKLLLARNLRIKGKASTIRAVCI

NKAGKDEKRSLGIPIIEDRVLQMLVKLALEPEWEAKFEPNSYGFRPGRNSHDAIRAVF

NQSRHKSQFVLDADIRKCFETISHQKLLKKLKTFPLIENQMKAWLTAGIMEKYSLLGS

DKSIPNPQGTPQGSVISPLLANIALHGMEDLTKEYYSKNFYKGPSKTTLKDRKSQIGL

IRYADDFVVLHKDEQVVHAIKALLSTWLYKHMGLELSSAKTSIKSTDNGYEFLGFHIT

SLNVKEKNKPKCQISVSKTSKKKFLDKTRRIMQNNRAIAAGHLIILLSPIIIGWSNYF

KYAECSTSFGQVKYGLFGQVRAWVFRRKSLGLRSRTKLKEKYFPPNTTVKFNGKVHTG

NWIFTGTVLGRRGNLKQVFLPYPSWVLAKRWVKVKQKSSPFDGQDIYWSHRNIQYCNW

NKRTIRLVKLQNYNCPICQRIFMENDILEIDHIIPIALGGTDNIRNLQVVHDFCHVKK

STTDKKFIVEIRSPSLKI"

exon 12895..12929

/gene="psbD"

/number=3

intron 12930..15164

/gene="psbD"

/number=3

CDS 13482..15077

/note="group II intron reverse transcriptase/maturase"

/codon_start=1

/transl_table=11

/product="putative reverse transcriptase and intron

maturase"

/translation="MLYLEFLVFLIVYWKNYMRKYTLRFQSQEDILVAWQNFEILSLG

TWVINWSDISWKSCNDIVIQIRRRIYIKTVELNNFTDSANLPKFLILQLQLRSLQRKL

LFCQSNLLVSVRHVTQPLSCETITNINFYKSITLDCTESLRLLMFIKSCLNVLEWQRY

HFLLNQSSRINNSNLRIQVTVDHIFQYIVKNALEPEHTNIELYMLDKYSFRSSYKTIK

SVVQSCVTLQQPWVLSVNIKEFFEIISFDYINSKIFKFPFFSLVNYWLSCSFLDDGPL

CFLLLDILLSGLGPEIKSQLFYFSYRHNSSLVNYLKDYIPSYTYVRYLDSVILLCETE

LKIKICRQILRDILNSRGLKLLVTKKIQFSHLSFGFDYLGVTVRIYCHYNIKSFRYQL

LIKPSSSSITKVKYKIRSLFIQYRSYHVSTLLKKMNPLIKEWGLYYSKFYSQETFSNL

DFYLYVLQSRYGGRNHPKKSRTWIINKYFGCFNLNRTDKWVFGIYSHGKVSYMEKFIW

IPGIQEEIGFFACSSLENKFCII"

exon 15165..15285

/gene="psbD"

/number=4

intron 15286..16103

/gene="psbD"

misc_feature 15544..15897

/note="potential group II twintron"

exon 16104..16126

/gene="psbD"

/number=5

intron 16127..16904

/gene="psbD"

/number=5

misc_feature 16351..16373

/note="3' motif for potential twintron"

exon 16905..17093

/gene="psbD"

/number=6

gene 17041..24400

/gene="psbC"

CDS join(17041..17194,19775..19887,20732..21350,22139..22239,

23269..23387,24175..24400)

/gene="psbC"

/codon_start=1

/transl_table=11

/product="photosystem II CP43 chlorophyll apoprotein"

/translation="MNNLYSLRRFFHVETLFNGTLTVGGRDQESTGFAWWSGNARLIN

LSGKLLGAHVAHAGLIVFWAGAMNLFEVAHFVPEKPMYEQGLILLISSAVLGFGGVYH

SIVGPETLEESFPFFGYVWKDKNKMTTILGIHLCLLGFGAYLLVWKAMYLGGIYDTWA

PGGGDVRVITNPTLSPFVIFGYILKSPFGGDGWIPSVDNMEDVIGGHIWIGTLEILGG

IWHILTKPFAWARRAFVWSGEAYLSYSLAAVSLMGLIAVPMVWFNTTVYPSEFFGPTG

PEASQSQAFTFLVRDQRLGANVASAQGPTGLGKYLMRSPTGEIIFGGETMRFWDFRGP

WLEPLRGPNGLDLNKLRNDIQPWQERRAAEYMTHAPLGSLNSVGGVATEINAVNYVNP

RSWLSTSHFVLGFFFFIGHLWHAGRARAAAAGFEKGLDRNFEAALFMRPLD"

exon 17041..17194

/gene="psbC"

/number=1

intron 17195..19774

/gene="psbC"

/number=1

CDS 17797..19185

/gene="psbC"

/codon_start=1

/transl_table=11

/product="ycf13"

/translation="MVKYNKKSLDLWKYLSWETCSYNLYRLQNRMFKSLVLGDIRTVL

QVQKLIMRSNSARFLAIREVTQVNVDKKISGIDGKISLTFTERFELNEYLRLNANNWK

PQVLKMIPNLRKDGTTKFLKVATIADRTWQCLIIFMLEPLYQAQVRPYICGVSSPYSY

FELQKFLFINLNVKAHGIQKRVLEVNLTGCFHSINLGLLMHKAIAPRGVKLGLYRTLQ

IGFTPEFSKDIFLSRLGLNSLLSDIALNGIESIHPCIRYADTVIFILKPLDSGKVIVE

KLNSFLINIGLDISCKKVKFLSSLMGFNFLGWHFRVFSNGNFRCTPSVENYKTFKRKI

KHIVNNSNYGSFIKATKLSPIVKNWRFYHKFCNMEGSRNSLFYMQRRAFKVFNKEAKQ

DRYSSKKLLDKAFPTLRKFDEEIFKTILESSSYHGHLKLKINSTDGHFSQGRDKFLLR

VQNHRCIHCGVM"

exon 19775..19887

/gene="psbC"

/number=2

intron 19888..20731

/gene="psbC"

/number=2

misc_feature 20146..20499

/gene="psbC"

/note="potential group II intron"

exon 20732..21350

/gene="psbC"

/number=3

intron 21351..22138

/gene="psbC"

/number=3

misc_feature 21575..21597

/gene="psbC"

/note="3' motif for potential twintron"

exon 22139..22239

/gene="psbC"

/number=4

intron 22240..23268

/gene="psbC"

/number=4

misc_feature 22385..22407

/gene="psbC"

/note="3' motif for potential twintron"

misc_feature 22406..22426

/gene="psbC"

/note="3' motif for potential twintron"

exon 23269..23387

/gene="psbC"

/number=5

intron 23388..24174

/gene="psbC"

/number=5

misc_feature 23612..23634

/gene="psbC"

/note="3' motif for potential twintron"

exon 24175..24400

/gene="psbC"

/number=6

gene 24506..24587

/gene="trnL"

tRNA 24506..24587

/gene="trnL"

/product="tRNA-Leu"

/anticodon=(pos:24539..24541,aa:Leu,seq:taa)

gene 25123..25467

/gene="rpl20"

CDS 25123..25467

/gene="rpl20"

/codon_start=1

/transl_table=11

/product="ribosomal protein L20"

/translation="MSRVKRGSVARKHRKKVLKFNKGFVGAHSTLYRPANQQSMKAFR

YSYIDRRKRKRDFRKLWIRRINAASRIYGFSYNEFINKLKKSKVVLNRKVLSQIAILD

VKSFKYLVDAVA"

gene 25511..26347

/gene="rps12"

CDS join(25511..25872,26317..26347)

/gene="rps12"

/codon_start=1

/transl_table=11

/product="ribosomal protein S12"

/translation="MPTIQQLIRFQRKKLQKKTKSPALKLCPQRRGVCTRVYTTTPKK

PNSALRKVARVRLSSGFEVTAYIPGIGHNLQEHSVVLIRGGRVKDLPGVRYHVVRGSL

DAAGVKNRTQGRSKYGVKRRRKQTYKAA"

exon 25511..25872

/gene="rps12"

/number=1

intron 25873..26316

/gene="rps12"

/number=1

exon 26317..26347

/gene="rps12"

/number=2

gene 26787..28046

/gene="rps7"

CDS join(26787..27047,27837..28046)

/gene="rps7"

/codon_start=1

/transl_table=11

/product="ribosomal protein S7"

/translation="MSRRRIAKKRQINPDPIYNSTLVTMLINRLLLKGKKTVAQQIFY

QAMKKIEESTQQDPLEVLRQAIVNVTPLVEVKARRVGGSTYQVPLEVKAERGTSLALR

WLIKSARTRSGREMVSKLSNEIIDAFNNTGAAVRKREETHRMAEANKAYAQFRF"

exon 26787..27047

/gene="rps7"

/number=1

intron 27048..27836

/gene="rps7"

/number=1

misc_feature 27272..27294

/gene="rps7"

/note="3' motif for potential twintron"

exon 27837..28046

/gene="rps7"

/number=2

gene 28132..31190

/gene="tufA"

CDS join(28132..28206,30036..31190)

/gene="tufA"

/codon_start=1

/transl_table=11

/product="translation elongation factor EF-Tu"

/translation="MAREKFERTKPHVNIGTIGHVDHGKTTLTAAITMTLATQGNSSA

KGYADIDSSPEEKARGITINTTHVEYETEARHYAHVDCPGHADYVKNMITGAAQMDGA

ILVVSGADGPMPQTKEHILLAKQVGVPNIVVFLNKEDQVDDEELLELVELEVRETLSA

YEFPGDDIPVIAGSALLSVEALTANPEIKKGENKWVDKIFNLMDKVDSYIPTPERDTD

KDFLMAVEDVFSITGRGTVATGRVERGVVNVGETVELVGLKDTKQTTVTGLEMFQKSL

DSALAGDNCGILLRGIQKADIERGMVIAKPGTINPHTKFDSQVYILTKEEGGRHTPFF

EGYRPQFYVRTTDVTGKIESFLADDGSRVQMVMPGDRIKMEVELIQPIAIEKGMRFAI

REGGRTVGAGVVLNIIA"

exon 28132..28206

/gene="tufA"

/number=1

intron 28207..30035

/gene="tufA"

/number=1

misc_feature 28329..28350

/gene="tufA"

/note="3' motif for potential twintron"

misc_feature 28398..28418

/gene="tufA"

/note="3' motif for potential twintron"

misc_feature 28986..29008

/gene="tufA"

/note="3' motif for potential twintron"

misc_feature 29275..29299

/gene="tufA"

/note="3' motif for potential twintron"

exon 30036..31190

/gene="tufA"

/number=2

gene 31270..31863

/gene="ycf4"

CDS 31270..31863

/gene="ycf4"

/codon_start=1

/transl_table=11

/product="photosystem I assembly protein ycf4"

/translation="MINFLNLSQGDIFRDENTFREKIIGSRRISNYFWATVIFLAASG

FFIVGLSSYLQFNLVFFLDAKEIVFFPQGIVMCFYGVAGILLSIYQWLVILWKVGEGF

NEFDKNKGIMRLFRWGFPGKNREIDLVYSLEDVEAIRVEIKEGINPSRTIFIRIKGKP

NIPLTQVGAPLPLSEIEKKASELASFLKVSIEGLVDK"

gene 31977..32048

/gene="trnQ"

tRNA 31977..32048

/gene="trnQ"

/product="tRNA-Gln"

/anticodon=(pos:32009..32011,aa:Gln,seq:ttg)

gene 32071..32157

/gene="trnS"

tRNA 32071..32157

/gene="trnS"

/product="tRNA-Ser"

/anticodon=(pos:32105..32107,aa:Ser,seq:gct)

gene complement(32424..32497)

/gene="trnM"

tRNA complement(32424..32497)

/gene="trnM"

/product="tRNA-Met"

/anticodon=(pos:complement(32461..32463),aa:Met,seq:cat)

gene complement(32517..32588)

/gene="trnG"

tRNA complement(32517..32588)

/gene="trnG"

/product="tRNA-Gly"

/anticodon=(pos:complement(32554..32556),aa:Gly,seq:gcc)

gene complement(32595..32667)

/gene="trnT"

tRNA complement(32595..32667)

/gene="trnT"

/product="tRNA-Thr"

/anticodon=(pos:complement(32632..32634),aa:Thr,seq:tgt)

gene 33935..35852

/gene="psbK"

CDS join(33935..33967,34472..34526,35782..35852)

/gene="psbK"

/codon_start=1

/transl_table=11

/product="photosystem II 3.9 kDa protein"

/translation="MFNMNIEILFSLLLARLPEAYAPFDPIVDVLPIIPVLFFLLAFV

WQASVSFR"

exon 33935..33967

/gene="psbK"

/number=1

intron 33968..34471

/gene="psbK"

/number=1

exon 34472..34526

/gene="psbK"

/number=2

intron 34527..35781

/gene="psbK"

/number=2

misc_feature 35589..35612

/gene="psbK"

/note="3' motif for potential twintron"

exon 35782..35852

/gene="psbK"

/number=3

gene 35933..36034

/gene="psb30"

CDS 35933..36034

/gene="psb30"

/codon_start=1

/transl_table=11

/product="photosystem II reaction center protein"

/translation="MNFELIAQLGSLLLIVIAGPLVVVLLSARQGNL"

gene 36815..36910

/gene="psaM"

CDS 36815..36910

/gene="psaM"

/codon_start=1

/transl_table=11

/product="photosystem I M-polypeptide"

/translation="MGITSSQVFIALITALIPGFFTIRLVKELYK"

gene 37176..37247

/gene="trnR"

tRNA 37176..37247

/gene="trnR"

/product="tRNA-Arg"

/anticodon=(pos:37208..37210,aa:Arg,seq:tct)

gene 37455..37568

/gene="psbI"

CDS 37455..37568

/gene="psbI"

/codon_start=1

/transl_table=11

/product="photosystem II I polypeptide"

/translation="MLTLKIFVYTVVIFFISLFIFGFLSNDPGRNPNAKDM"

gene 37641..37714

/gene="trnD"

tRNA 37641..37714

/gene="trnD"

/product="tRNA-Asp"

/anticodon=(pos:37675..37677,aa:Asp,seq:gtc)

gene 37815..39506

/gene="petG"

CDS join(37815..37859,39438..39506)

/gene="petG"

/codon_start=1

/transl_table=11

/product="cytochrome b6/f complex subunit V"

/translation="MVEPLLSGIVLGLIPSSIAGLFVTAYLQYRRGEQLTF"

exon 37815..37859

/gene="petG"

/number=1

intron 37860..39437

/gene="petG"

/number=1

exon 39438..39506

/gene="petG"

/number=2

gene 39571..39643

/gene="trnK"

tRNA 39571..39643

/gene="trnK"

/product="tRNA-Lys"

/anticodon=(pos:39604..39606,aa:Lys,seq:ttt)

gene 39689..39761

/gene="trnF"

tRNA 39689..39761

/gene="trnF"

/product="tRNA-Phe"

/anticodon=(pos:39722..39724,aa:Phe,seq:gaa)

gene 39816..47985

/gene="psaA"

CDS join(39816..40158,40986..41469,42244..42792,43616..43863,

44673..44845,46754..46962,47736..47985)

/gene="psaA"

/codon_start=1

/transl_table=11

/product="photosystem I P700 apoprotein A1"

/translation="MTISPPEREIKKVQVVVDRNPVETSFEKWAKPGHFSRTLSKGPA

TTTWIWNLHADAHDFDSHTTDLEDISRKVFSAHFGQLAIIEIWISGMFFHGARFSNYE

AWVMDPTHIKPSAQVVWPVVGQEILNGDVGGGFQGIQITSGFFQIWRASGITSELQLY

STAIAGLIMAALLFFAGWFHYHKAAPKLEWFQNVESMLNHHLAGLLGLGCLSWAGHQI

HISLPINKLLDAGVDAKEIPLPHEFMINRDLMAQLYPSFAKGLTPFFTLHWSEYSDFL

TFRGGLNPVTGGLWLSDTAHHHLALAVLFIVAGHMYRTNWGIGHSMKEILETHKGPFT

GQGHKGLYEIFTTSWHAQLSLNLATLGSLSIIVAQHMYAMPPYPYLATDYGTQLSIFT

HHMWIGAFCIVGAAAHAAIFMVRDYDPTVNYNNLLDRVLRHRDSIISHLNWVCIFLGL

HSFGLYIHNDTMSALGRPQDMFSDTAIQLQPVFAQWVQNTHYLAPNLTAPNADLATSA

VWGGDIVTVGGKVAMMPIPLGTADFMVHHIHAFTIHVTVLILLKGVLFARSSRLIPDK

ANLGFRFPCDGPGRGGTCQVSAWDHVFLGLFWMYNSISVVIFHFSWKMQSDVWGTVTA

NGISHITGGNFAQSAITINGWLRDFLWAQASQVIQSYGSALSAYGLIFLGAHFIWAFS

LMFLFSGRGYWQELIESIVWAHNKLKVAPAIQPRALSITQGRAVGVAHYLLGGIATTW

SFFLARIIAVG"

exon 39816..40158

/gene="psaA"

/number=1

intron 40159..40985

/gene="psaA"

/number=1

exon 40986..41469

/gene="psaA"

intron 41470..42243

/gene="psaA"

/number=2

exon 42244..42792

/gene="psaA"

/number=3

intron 42793..43615

/gene="psaA"

/number=3

exon 43616..43863

/gene="psaA"

/number=4

intron 43864..44672

/gene="psaA"

/number=4

exon 44673..44845

/gene="psaA"

/number=5

intron 44846..46753

/gene="psaA"

/number=5

misc_feature 45046..45070

/gene="psaA"

/note="3' motif for potential twintron"

CDS 45318..46634

/gene="psaA"

/note="group II intron reverse transcriptase/maturase"

/codon_start=1

/transl_table=11

/product="putative reverse transcriptase and intron

maturase"

/translation="MVNVNWETVNWRLVQNRVSRIQYRIYEQKKRFKRYYLQHVLANS

FETKFLALELTLQNYEVAVPLKSKRDIALQITFETTTNVVDTKVNVEFMTLLRFAIQR

FLFYILVPYYYASSQFKTFIPFYRPIPHDIIKKIKKVLLKNNYQYAVQINLNFNYISN

IVLLSVVQRLLVSPLLGSSIKPYLRYYQRHVSPRPKQYWKKISLPYYEPLKILFCDLL

VYELLNQIQQLDNELNLILVPGRDKLVIFHLNLKVLKTILPSISKLSKKDICVYLHRK

DLKIIKLSVGFSLYNFQIILLQTSNLTRKIHIFPGKKSQDSYLRKFSQICHKNRNVSA

YILITKLRPLVLKWSMYFQYSNCIKVFIKVTYKSNQILRAWVFRRDNKNGRNVIKEKY

FPSGNTYNFPRMSHKNNWVLCGEFNKNNLYKKKVFLPQLHWAPGLK"

misc_feature 46253..46277

/gene="psaA"

/note="3' motif for potential twintron"

misc_feature 46664..46686

/gene="psaA"

/note="3' motif for potential twintron"

misc_feature 46665..46685

/gene="psaA"

/note="3' motif for potential twintron"

exon 46754..46962

/gene="psaA"

/number=6

intron 46963..47735

/gene="psaA"

/number=6

exon 47736..47985

/gene="psaA"

/number=7

gene 48102..51873

/gene="psaB"

CDS join(48102..48770,49557..50873,51655..51873)

/gene="psaB"

/codon_start=1

/transl_table=11

/product="photosystem I P700 apoprotein A2"

/translation="MATKFPKFSKGLAQDPTTRRIWFGIATAHDFESHDGMTEENLYQ

KIFASHFGQLAIIFLWTSGNLFHVAWQGNFEQWVTDPLHIRPIAHAIWDPHFGQPAIE

AFTRSNATGPVNIAFSGVYHWWYTIGMRTNNDLYNGSIFLLFVSALALFAGWLHLQPN

YKPKVSWFKNAESRLNHHLSGLIGVSSLAWTGHLIHVAIPESRGQHVRWDNFLSTSPH

PAGLGPFFSGNWSVYAQNPDSQSHIFGTSEGAGTAILTFLGGFHPETKSLWLTDMSHH

HLALAVLFIIAGHMYRTNFGIGHNMQEILEAHNPPSGKLGAGHKGLYDTVNESLHFQL

GLALACVGVAASLTAQHMYSLPAYAFMSQDYTTMAALYTHHQYIAGFVMSGAFAHGAI

FFIRDYDPEQNKGNVLARMLEHKEAIISHLSWVSLFLGFHTLGLYVHNDAMQAFGTPE

KQILIEPIFAQWIQAAQGKALYGFDVILSANSNTAISASETIWLPGWLSAINDSTNTL

FLPIGPGDFLVHHAIALGLHTTTLILVKGALDARGSKLMPDKKDFGYSFPCDGPGRGG

TCDISAWDAFYLAVFWMLNTIGWTTFYWHWKHITLWQGNVSQFDESSTYIMGWLRDYL

WLNSSQLINGYNPFGMNSLAVWGWMFLFGHLVWATGFMFLISWRGYWQELIETLAWAH

ERTPFATAVQWKDKPVALSIVQARLVGLAHFCVGYVFTYAAFLIASTSGKFG"

exon 48102..48770

/gene="psaB"

/number=1

intron 48771..49556

/gene="psaB"

/number=1

exon 49557..50873

/gene="psaB"

/number=2

intron 50874..51654

/gene="psaB"

/number=2

exon 51655..51873

/gene="psaB"

/number=3

gene 52072..52317

/gene="psbE"

CDS 52072..52317

/gene="psbE"

/codon_start=1

/transl_table=11

/product="photosystem II cytochrome b559 alpha subunit"

/translation="MAGSTGERPFSDIITSIRYWVIHSVTIPSLFVAGWIFVSTGLAY

DVFGTPRPNEYFTETRKETPLISDRFGALEQMDQLIK"

gene 52327..52449

/gene="psbF"

CDS 52327..52449

/gene="psbF"

/codon_start=1

/transl_table=11

/product="photosystem II cytochrome b559 beta subunit"

/translation="MTTNKFTYPIFTFRWLTIHALAVPTVFFIGAISSMQFIQR"

gene 52994..53110

/gene="psbL"

CDS 52994..53110

/gene="psbL"

/codon_start=1

/transl_table=11

/product="photosystem II L protein"

/translation="MTQPNPNKQSVELNRTSLYWGLLLIFVLAVLFSSYIFN"

gene 53186..53314

/gene="psbJ"

CDS 53186..53314

/gene="psbJ"

/codon_start=1

/transl_table=11

/product="photosystem II J protein"

/translation="MSNSGTTGRIPLWFVGTIAGTAAIGILALFFYGSYSGLGSSL"

gene 53422..53532

/gene="psaI"

CDS 53422..53532

/gene="psaI"

/codon_start=1

/transl_table=11

/product="photosystem I 4 kDa polypeptide subunit"

/translation="MSASFLPSIFVPLIGLVLPVLFVTSFFIYVGKDQIN"

gene complement(54072..54153)

/gene="trnY"

tRNA complement(54072..54153)

/gene="trnY"

/product="tRNA-Tyr"

/anticodon=(pos:complement(54117..54119),aa:Tyr,seq:gta)

gene 54377..54988

/gene="rps4"

CDS 54377..54988

/gene="rps4"

/codon_start=1

/transl_table=11

/product="ribosomal protein S4"

/translation="MSRYRGPRLRIVRRLGELPGLTTKTSKKLNPPGQHGASSKQKLS

QYSIRLREKQKLRYNYGVTERQLLNYVKKSRKGKGSSGKILLTLLEMRLDNTAYRLRL

APTIVGARQLVSHGHILVNGKSVNIPSYQCQPRDILSVKNSKSSKGLVEKNLEALGNV

VIPDHLSFNKDQLEGKVNNIIDRKSVALIINELLVVEYYSRKV"

gene 55036..55428

/gene="rps11"

CDS 55036..55428

/gene="rps11"

/codon_start=1

/transl_table=11

/product="ribosomal protein S11"

/translation="MARQQKKTGVRNIKRKVSKGVVHIKATFNNTIVSITDPKGEVIS

WSSAGACGFKGARKGTPFAAQTAAENAAKKSLDQGMRQVEIIVTGPGAGRETAIRSIQ

TAGLGISIIRDITPVPHNGCRPPKKRRV"

gene 55633..56427

/gene="rpoA"

CDS 55633..56427

/gene="rpoA"

/codon_start=1

/transl_table=11

/product="RNA polymerase alpha subunit"

/translation="MYQPKLSKLLTIECIEHKVSEANSRYGRFKIGPLNTGQGITVGN

SLRRTLLSDITGLAITNAKINVENISGISELVESDSELIIPKFHEFSTIPGVRESVLE

ILLNLKNIILSDNSTDFAEDQLGSLQCTGPCQITARDLILPETIKVVDPDQYIATMVS

NQIFSIKVIIKHGRGVTEIPVQLDLKTQQKDCFDFLTIDATYIPIKKVNYTIEEKFLG

DGIDSLKELITLEVWTNGSVKPQKAVSSAINTLVDLYLSIQNTMVV"

gene 56940..57338

/gene="rps9"

CDS 56940..57338

/gene="rps9"

/codon_start=1

/transl_table=11

/product="ribosomal protein S9"

/translation="MAPTTQYKSTGRRKSAVAQVILIPGSGKIIINGKDGTEYMQYNP

RLVSTIQSPLNLLSLEEGYDVLVRSSGGGLSGQAEAIRLGIARALCKLDVTNRGPLKS

EGFLTRNALCVERKKYGLKKARKAPQYSKR"

CDS 57647..58042

/codon_start=1

/transl_table=11

/product="ribosomal protein L12"

/translation="MSIKTDEIIEQLKGITLLEASELVAQIEETFGVDASAPTGGMMV

AAMPGLGGGAAEAVEEKTEFDVVIEEVPSAKRITVIKVVRNLTSLGLKEAKGLIESTP

KAVREGVSKEEAEDAKKQLEEAGATVSIK"

CDS 58960..59154

/codon_start=1

/transl_table=11

/product="photosystem II protein Z"

/translation="MDIILVFQFSLLALIAVSLLMVIAVPVVFATPDGWNGNKQYVLT

GAAAWAGLVFVVGALNSVVV"

gene complement(59252..59336)

/gene="trnS"

tRNA complement(59252..59336)

/gene="trnS"

/product="tRNA-Ser"

/anticodon=(pos:complement(59300..59302),aa:Ser,seq:tga)

gene complement(59368..59441)

/gene="trnP"

tRNA complement(59368..59441)

/gene="trnP"

/product="tRNA-Pro"

/anticodon=(pos:complement(59405..59407),aa:Pro,seq:tgg)

gene 59548..59673

/gene="psaJ"

CDS 59548..59673

/gene="psaJ"

/codon_start=1

/transl_table=11

/product="photosystem I 5kDa protein"

/translation="MKDFTTYLSTAPVIATIWFGLLAGLLIEINRFFPDALTFAF"

CDS complement(59877..61379)

/note="group II intron reverse transcriptase/maturase"

/codon_start=1

/transl_table=11

/product="putative reverse transcriptase and intron

maturase"

/translation="MLNIHMSKAITNNWSNVSWRFLRGKVFNIQCRIYQATKANKKRT

TRNLQKVLLSSKSAKFVAMYRTTEIFSNDKSTNPNISNLIKLDNKMTENIISAVRNGS

HSKHQLLLESTKQYLVRLVLEPEWESFFTNNEIASFGFRPGYCVHDAIQSILNQICSS

HPYVFSTNIIRLDNKVNSTHLLQFLHLDDFPLLQAQIESWLNGGCLDKRDFLYCGNKN

YSTLSFLLVNIILSRIPSVIKLAQLDFQNFNNGKTSANISIVQYGDSLVFSYPEINGL

IHIKKSLDKFLLPIGLIMDQSEYGITHITNYRTHSKPGFNFLGVYIRKYRSITGRYKL

VVTPKFSKVKEHLCHIRQVVRSNKSSPARRIIQLLNPIIQGWAYYYHYTNPTKIFNFC

DFRIRIILRRWMVYRHPMKSWQWLRKKYFVYLSKDTHNIIKSDRFFDNNFRSNSLSYT

FNSIILNRHTETYLSTWTNVDKRRSFFDGDFCYWRIKLQNFPELDHRKLI"

gene complement(61586..61801)

/gene="rps18"

CDS complement(61586..61801)

/gene="rps18"

/codon_start=1

/transl_table=11

/product="ribosomal protein S18"

/translation="MAISYNRTSSIKPSEVIDYKNIDLLRKFITVQGKILPRRITKLT

AKQQRAVTSSIKQARTLGLLPFINKES"

gene complement(62185..63696)

/gene="atpA"

CDS complement(62185..63696)

/gene="atpA"

/codon_start=1

/transl_table=11

/product="ATPase alpha subunit"

/translation="MVKIRPDEISSIIRQQIEQYNQEVKVVNVGTVLQVGDGIARIYG

LEKVMAGELVEFDEGTVGIALNLEADNVGAVLMGDGFKIQEGSSVKATGKIAQIAVGE

NYLGRIVDALARPIDGKGEIASSETRLIESPAPGIIARRSVYEPLQTGLVAIDAMIPI

GRGQRELIIGDRQTGKTAVATDTILNQQGQGVICVYVAIGQKASSVAQIVNTLEERGA

MDYTIVVAENADAPATLQYLAPYTGAALAEFFMYTGRHTLVIYDDLSKQAQAYRQMSL

LLRRPPGREAYPGDVFYLHSRLLERAAKLSDELGEGSMTALPIVETQAGDVSAYIPTN

VISITDGQIFLSSDIFNSGIRPAINVGISVSRVGSAAQIKAMKQVAGKLKLELAQFAE

LEAFAQFSSDLDATTQAQLARGQRLRELLKQSQSSPLVVADQVATIFTGVNGFLDDIA

VEDVPAFLTGLREYINTNKPNYAEIINSTKAFTSEAEGIVKSAISEYKAVFSK"

gene complement(63796..65155)

/gene="atpF"

CDS complement(join(63796..64320,65135..65155))

/gene="atpF"

/codon_start=1

/transl_table=11

/product="ATPase subunit I"

/translation="MIVSNINNTFASVAESEGFGINTDLFETNILNLAVVVGVLVYFG

KDILSDTLKTRKEAILKSLQDADNKFQEATDKLNDAKKQFDFAKIRAEEITAQGLVTA

EKSSNKLLARVEDDIKRLEDGKVATLRFEEEKAVTEVCEKVSRLALDQAVDNLNKRLN

PALQQRIIQLNLALLRNLVTK"

exon complement(63796..64320)

/gene="atpF"

/number=2

intron complement(64321..65134)

/gene="atpF"

/number=1

misc_feature complement(64928..64950)

/gene="atpF"

/note="3' motif for potential twintron"

exon complement(65135..65155)

/gene="atpF"

/number=1

gene complement(65239..66262)

/gene="atpH"

CDS complement(join(65239..65399,66175..66262))

/gene="atpH"

/codon_start=1

/transl_table=11

/product="ATPase subunit III"

/translation="MNPIICGASVIGAGLAIGLGAIGPGIGQGTAAGAAVEGIARQPA

QEGKIRGTLLLSLAFMEALTIYGLVVALAIIFANPFVG"

exon complement(65239..65399)

/gene="atpH"

/number=2

intron complement(65400..66174)

/gene="atpH"

/number=1

exon complement(66175..66262)

/gene="atpH"

/number=1

gene complement(66547..67275)

/gene="atpI"

CDS complement(66547..67275)

/gene="atpI"

/codon_start=1

/transl_table=11

/product="ATPase subunit IV"

/translation="MDAIVFNSIYEIASVEVGQHFYWSLGDYEVHGQVLINSWIVIGV

IILGGTLTTRELKTIPESGQNFAEILTEFIRDIAKTQIGEKEYKKWVPYLGTLFLFIF

VANWSGALIPWKIIELPNGELGAPTNDINTTAGLALLTSAAYFYAGLGKRGLEFFTKY

IEPTPVLLPINVLEDFTKPLSLAFRLFGNILADELVVGVLVSLVPLIVPIPLIFLGLF

TSGIQALIFATLSGAYIGEAMEGH"

gene complement(67341..69433)

/gene="rps2"

CDS complement(join(67341..67600,68522..68758,69250..69433))

/gene="rps2"

/codon_start=1

/transl_table=11

/product="ribosomal protein S2"

/translation="MITVKQMVDSGVHLGHQVRRWNPKMASYIYGERNGIHIIDILQT

LICLSDTTDYLVKAGKENKTILFVGTKRQFASIIESCAEKSNSHYVNQRWLGGMLTNW

STMKVCIENLQILNQQEEDGTLSRLTKKEAAILNKRKDKLEKYFGGVKEMGSLPDVVI

LVGQPRELIAVKECIKLGIKTITITDTNCDPTLTDFAIPGNDDSIRSVELILNELTDS

IVKGQNTK"

exon complement(67341..67600)

/gene="rps2"

/number=3

intron complement(67601..68521)

/gene="rps2"

/number=2

misc_feature complement(67786..68297)

/gene="rps2"

/note="potential group II twintron"

exon complement(68522..68758)

/gene="rps2"

/number=2

intron complement(68759..69249)

/gene="rps2"

/number=1

exon complement(69250..69433)

/gene="rps2"

/number=1

gene complement(69699..69770)

/gene="trnC"

tRNA complement(69699..69770)

/gene="trnC"

/product="tRNA-Cys"

/anticodon=(pos:complement(69736..69738),aa:Cys,seq:gca)

gene 70830..77040

/gene="rpoB"

CDS join(70830..71384,72251..73379,74492..75352,76127..77040)

/gene="rpoB"

/codon_start=1

/transl_table=11

/product="RNA polymerase beta subunit"

/translation="MIKNFVLPDLIEIQRASFISFLEKGLSEELENISSIKTEDGKLE

LILHTSKLKFKRPKFSPQDAIKKAQTYSAALYVPAQINYNNRFKSKLQNICFGEIPLM

TERGTFIINGSPRVIVNQIVRSPGIYYKAEFNKENQKTFTGTIISNRGSWFRIETDKD

GYVWAKIDKVKKIPIFILLQAFGLPQKKIFYAVRHPEFLVKSLEKGNPTTTFEALILL

HSLLRPDKQATVKDARNLIYSNFMDPKRYDLGEVGRLKLNKKLKISTSPKCRSLRPED

ILAAIDYLINLDFGIGSIDDIDDLKNRRIRSSGELIQNQIRIGISRVERITHEKLEKF

QKKENITNSQLLNKNSKLKTQLDNVHTPATLISSKPLISSLREFFGSSQLSQFMDETN

SLSEITHKRRITSFGPGGLNRERAGLAVREIHPSHYGRICPIETPEGPNAGLVGSITT

YARINKHGFIESPFYKVRDKIVNRNVSPFFLSAEQEENAALAPGDLLISSNGELQIRD

KLIPIRYKQEFTTALPENVNYMAISPIQMISVATALIPFLEHDDANRALMGSNMQRQA

VPVINSQRPVVGTGLELQTARDCGSTIIAKKSGFVQDVSAEKIIIDSSPTYLRNFTDH

TNKYKNFSKSLRFQSLITEPIKRHKKFIKFKDLSNLFLDKKISIDDHLLKLIANTKKD

KIKKRVNEKNLDIYKLQKYQRSNQDTCINQKPLVHQGEWVKKGDILADGASTSKGELA

LGQNILIAYMPWEGYNFEDAILISERLVQDNVYTSIHIEKYEVQIQQTKLGPEETTKN

IPNISNLATRYLDKNGIIIPGTWVESGDILVGKVTPKGESDQTPEGRLLRAIFGEKGR

DVKNTSLKVPNGVKGRVIDIQIFQKTSIHVYLAQKRKIQVGDKIAGRHGNKGIISNIL

PCQDMPYLQDGTPVDMVLNPLGVPSRMNVGQVLECLLGLAGKNLDENYKIVPFDEMHG

NETSRNLVYNKLYEARKKTGHKWLFDPNSPGKSRLFDGRTGESFEQPITVGYAYMLKL

VHLVDDKIHARSTGPYSLVTQQPLGGRAKNGGQRLGEMEVWALEGFGAAYTLQELLTV

KSDDIKGRNDALNAIIKGKTIPNPGTPESFKVLIRELQSLCLDIGLYNIDKFNNTKEI

DIIHMK"

exon 70830..71384

/gene="rpoB"

/number=1

intron 71385..72250

/gene="rpoB"

/number=1

misc_feature 71645..72000

/gene="rpoB"

/note="potential group II twintron"

exon 72251..73379

/gene="rpoB"

/number=2

intron 73380..74491

/gene="rpoB"

/number=2

misc_feature 73826..73849

/gene="rpoB"

/note="3' motif for potential twintron"

exon 74492..75352

/gene="rpoB"

/number=3

intron 75353..76126

/gene="rpoB"

/number=3

misc_feature 75560..75584

/gene="rpoB"

/note="3' motif for potential twintron"

exon 76127..77040

/gene="rpoB"

/number=4

gene 77235..80176

/gene="rpoC1"

CDS join(77235..77246,77603..79479,80134..80176)

/gene="rpoC1"

/codon_start=1

/transl_table=11

/product="RNA polymerase beta' subunit"

/translation="MIEYEYIRIHLASPERVRQWSERLLPNGTKIGEVTKSETINYRT

FKPEMDGLFCERIFGPVKDWECHCGRYKRARHRQNGVNTIICSQCGVEVTESRVRRYR

LGYIQLASPVTHVWYLKSIPSYIAILLDKPIKEIEKIVYFTGYLAINPDSINYTIPPG

NDWNYYRWYYSKSFFNGNKKPQENLKTAQGTLLDNIIGAQAIQNLLSEINLEYTAQNI

REDLKSIQKTDALTEIELGKIFRKKKKHVRRLRLINYFIQTKSKPEWMVLSCLPVLPP

DLRPMVQLDGGRFATSDLNDLYRRVINRNNRLLRLTKMLAPEILIRNEKRLLQESVDA

LIDNGKRGKAVAGTNNRPLKSLSDIIEGKQGRFRQNLLGKRVDYSGRSVIVVGPKLKL

HECGLPKEMAIELFQPFVIHKLIQLRLANNIRGAKIKIQQNEPVIWEILEKVIKGHPV

LLNRAPTLHRLGIQAFQPKLVRGRAIQLHPLVCPAFNADFDGDQMAVHVPLSLEAQAE

ARILMLASNNWTSPATGHPILVPSQDMILGCYFLTIENLSLYNLLQEIKYFTGLNDAL

LAYEQKKIGLHSFIWIRSNLKEYTKNSIEIKIENNSIILKKEHENITIEGNKKQNLVQ

YIRTTTGRLLFNQVIKEFL"

exon 77235..77246

/gene="rpoC1"

/number=1

intron 77247..77602

/gene="rpoC1"

/number=1

misc_feature 77448..77467

/gene="rpoC1"

/note="3' motif for potential twintron"

exon 77603..79479

/gene="rpoC1"

/number=2

intron 79480..80133

/gene="rpoC1"

exon 80134..80176

/gene="rpoC1"

/number=3

gene 80269..83235

/gene="rpoC2"

CDS 80269..83235

/gene="rpoC2"

/codon_start=1

/transl_table=11

/product="RNA polymerase beta'' subunit"

/translation="MESFFCNRTFDKGEMRKLIQWFLVNYGTARTAKLVDRLKTVGFH

YATKAGISLGIDDLKIPPIKTRLLMNAETKIEENEKHFLSGKITAVERLQKVIDVWNT

TNELLKNEVIIHFRQTDLLNPVYMMAFSGARGNISQVRQLVGMRGLMADSRGEIIDLP

IKSNFREGLNVTEYLISCYGARKGLVDTALRTANSGYLTRRLVDVAQSVIINKTDCGT

YHGILVNPLIKDKKTYISIEKRLIGRVLAKDVFDIQNKKLIASRGQDICSYLAPKILK

NTTQRLYVRSPLTCESNRGICQLCYGWSLAHGRLAQIGEAVGIIAAQSIGEPGTQLTM

RTFHTGGVFAGNVADRIYAPHDGILEYFSDSGGKKIRTRYGEDVFFTFTEKRLFIKET

ELNTSTLTLPRYTIIFIKPGQKIFARQIIAEMSSITEIVPSSDKNDWVEASKEVKTNI

GGQIYFDNLITVQQHNASEKIQQINKNQNVTKAKENGLVWVLNGNLFSFLTIFKKLSI

ESKQRYTLSEMKISKNRKFQIFIDNVCNKSIYKKNQNIKHRILQPCSPTFTNLETYNY

RNKQIHDKNYENPYRYQFYTKNLNIKINLSKVQQLKKQVKIDFHQNKVKAIRKNRLGK

DYKTYKKPIPHCETSYLEKYDNINFCSQDIKRYPKELSFDYQNLNKQKHCQYEEITVL

TSNKQCLIHNTENQVIFDTQLRTLKVNIGDLICKGNEIADSLRSTQSGQIISIQHNQI

ILRKGIPNLASENTLVGVNNRDLIAKNNLLFHLVYKKPKTGDIVQGLPKIEELLEARR

TKDLQTIVDNPHEKLRQQFNFYKQKYDNAIATRKSAEKIQQFLVNGIQLVYQSQGIEI

SDKHIEIIVKQMTSKVLIEEGGETSLLSGEVIEINRIEKINKNIIKKAEYQPILLGIT

KASLNTESFISAASFQETTRVLIQAAVEGKTDWLYGLKENVILGRLIPAGTGFQTKEK

STTKLLQTPTANKFDN"

gene 83453..83525

/gene="trnV"

tRNA 83453..83525

/gene="trnV"

/product="tRNA-Val"

/anticodon=(pos:83486..83488,aa:Val,seq:tac)

CDS 84175..84516

/codon_start=1

/transl_table=11

/product="hypothetical protein"

/translation="MKKIQRRFTGGDNPFKQPSLKKFGFRDIGVPFNFTLVKDIHATI

ECCLDIRKRFVNQEFLNFCYTNVDRVLTQEILNQTVVMRGEDIPSWLKPIEKRCLVCT

LLCLLNRPHRN"

gene complement(85824..85939)

/gene="rrn5"

rRNA complement(85824..85939)

/gene="rrn5"

/product="5S ribosomal RNA"

gene complement(86000..88870)

/gene="rrn23"

rRNA complement(86000..88870)

/gene="rrn23"

/product="23S ribosomal RNA"

gene complement(88925..88997)

/gene="trnA"

tRNA complement(88925..88997)

/gene="trnA"

/product="tRNA-Ala"

/anticodon=(pos:complement(88962..88964),aa:Ala,seq:tgc)

gene complement(89057..89130)

/gene="trnI"

tRNA complement(89057..89130)

/gene="trnI"

/product="tRNA-Ile"

/anticodon=(pos:complement(89094..89096),aa:Ile,seq:gat)

gene complement(89222..90705)

/gene="rrn16"

rRNA complement(89222..90705)

/gene="rrn16"

/product="16S ribosomal RNA"

gene complement(91544..92605)

/gene="psbA"

CDS complement(91544..92605)

/gene="psbA"

/codon_start=1

/transl_table=11

/product="photosystem II core 32 kDa protein"

/translation="MTATLERRESTSLWARFCSWITSTENRLYIGWFGVLMIPTLLTA

TSVFIIAFIAAPPVDIDGIREPVSGSLLYGNNIISGAVIPTSNAIGLHFYPVWEAASL

DEWLYNGGPYQLIVTHFFIGICCYMGREWELSFRLGMRPWIAVAYSAPVAAATAVFII

YPLGQGSFSDGMPLGISGTFNFMIVFQAEHNILMHPFHMLGVAGVFGGSLFSAMHGSL

VTSSLIRETTENESANAGYKFGQEEETYNIVAAHGYFGRLIFQYASFNNSRSLHFFLA

AWPVVGIWFTALGVSTMAFNLNGLNFNQSVVDSQGRVINTWADIINRANLGMEVMHER

NAHNFPLDLAAIEAPSVNG"

gene 92854..93159

/gene="ycf65"

CDS 92854..93159

/gene="ycf65"

/codon_start=1

/transl_table=11

/product="putative ribosomal protein 3"

/translation="MNKYVLKFLWLEKSIAVALDQQIGENTSPITEYFFWPRKDAWEE

LKTELEKKPWISQNEAIVLLNQTTEVINYWQEENGTNRRDLQKAKSKFPECLFIGHD"

gene complement(93219..94483)

/gene="rps14"

CDS complement(join(93219..93344,94307..94483))

/gene="rps14"

/codon_start=1

/transl_table=11

/product="ribosomal protein S14"

/translation="MAKKSMVEREAKRQKLVAKHNASREILKAKIKEADSFEAKLNYH

RQLQALPRNSAPSRLHNRCMVTGRPKGYYRFFGLSRHVLREMAHEGILPGVKKASW"

exon complement(93219..93344)

/gene="rps14"

/number=2

intron complement(93345..94306)

/gene="rps14"

/number=1

exon complement(94307..94483)

/gene="rps14"

/number=1

gene complement(94517..94600)

/gene="trnM"

tRNA complement(94517..94600)

/gene="trnM"

/product="tRNA-Met"

/anticodon=(pos:complement(94564..94566),aa:Met,seq:cat)

gene complement(94713..94826)

/gene="rpl36"

CDS complement(94713..94826)

/gene="rpl36"

/codon_start=1

/transl_table=11

/product="ribosomal protein L36"

/translation="MKVRASVRKICEKCRLIRRKTRVMVICSNPKHKQRQG"

gene complement(94993..95409)

/gene="rps8"

CDS complement(94993..95409)

/gene="rps8"

/codon_start=1

/transl_table=11

/product="ribosomal protein S8"

/translation="MVNDIVSDMLTRIRNANLVKAQIVLVPRTKMTVSVSDILKQEGF

IQSFEELDIPTKGQKYVNKFLVLCLKYKGPKQKPYITALKRISKPGLRVYVNQTHIPR

VLGGIGMAILSTSKGLMTDRQARINKLGGEVLCYIW"

gene complement(95487..96026)

/gene="rpl5"

CDS complement(95487..96026)

/gene="rpl5"

/codon_start=1

/transl_table=11

/product="ribosomal protein L5"

/translation="MQRLKKIYLKDVIPQLKEQFNYINLQQVPQLKKIVINRGFGENY

QNVKVLESSLDELTNISGQRGIITKSKKAIASFKVREGMPVGMTVTLRGDKMYGFLDR

LINLSLPRIRDFQGVSSKSFDSRGNYSIGFDDQLMFPEVDFDKVNQTQGMDITIVTSA

KTDPEGVALLKALGMPFKS"

gene complement(96085..96450)

/gene="rpl14"

CDS complement(96085..96450)

/gene="rpl14"

/codon_start=1

/transl_table=11

/product="ribosomal protein L14"

/translation="MIQPQTYLNVADNSGARKIMCIQVLGSNRQYASIGDVIVAVVKD

ATPNMPLKKSDVVKAVIVRTCKALRRESGMTIRFDDNAAVVVSAEGAPKGTRVFGPVA

RELRDKNFIKIVSLAPEVL"

gene complement(96521..96928)

/gene="rpl16"

CDS complement(96521..96928)

/gene="rpl16"

/codon_start=1

/transl_table=11

/product="ribosomal protein L16"

/translation="MLSPKRTKYRKYHRGNMRGKALRGNKLSFGEIGLQATEAGWITS

RQIEAARRVITRTARRGGKLWIRIFPDKPVTFRAAETRMGSGKGNVEYWVAVVKPGKV

LYEIKGVAELVARSAMKTASYKMPVKTRIISKL"

CDS complement(96992..97300)

/codon_start=1

/transl_table=11

/product="putative reverse transcriptase and intron

maturase"

/translation="MRLELDANKFACSVLRENAFLVLLNIIDWTNFYKDSDYFYVVCL

EVDIYLFRLLWKWVHKRHPSRSSIWLLKRYWCKKYGKFIFYNFAPTISRSYTLVFHCQ

"

CDS complement(98473..98985)

/codon_start=1

/transl_table=11

/product="putative reverse transcriptase and intron

maturase"

/translation="MSRIQRDLLVRIFGRFFKNLPSTFSGLDNFVKEKFRIQSRSIEL

LFPIFVRYFNTILLTGQNPVQLYMFRYIFSEFISIRGLRITVSTIPVYFCEGFTFLSW

YFYQTYNGVFFCTLSKSTLRFHKRLLKSIVKNSLALPVNVLIRQLNNQVRQAHYFVLP

VPNSGGFIST"

CDS complement(101296..101958)

/note="putative reverse transcriptase and intron maturase"

/codon_start=1

/transl_table=11

/product="hypothetical protein"

/translation="MLVRSLFSSIRNKNWLTIKWVIVERRVLRVQHLIVRATQKIDRR

TLRVFQKLLVRSFFSRLKSIRYVTQEDTKRFIPGGDGEIVVSNESKFSLALNFAINSN

VTKLRLIYFFTKECLYRSILVPTLRERVFQVLWNLALVPVVESTRDSLVYTFPRSQVT

DDYSKSVKNYFEFPYSLSWISTVSIEPIQESFKSIWLLCNAPMDRQVCKGWQRDNFLV

QI"

gene complement(102050..102694)

/gene="rps3"

CDS complement(102050..102694)

/gene="rps3"

/codon_start=1

/transl_table=11

/product="ribosomal protein S3"

/translation="MGQKVNPLGFRIGITQDHRSHWYAKPSDYSSLLMEDNFIRSYVN

SKLSEAGISLVKIQRKSDHLQIDIHAERPSIIIGRNNSGLDTFRQDLNNQLSRKFTLR

DIQLNIIEVTNPDSHSKLLAEFISQQLESRVPFRRVVRNATQRAQKAGVKGIKIQIAG

RLNGAEIARSEWVREGQVPLQTLRANIDYCSHKAHTIYGILGIKVWIYAPNVSS"

gene complement(102865..103218)

/gene="rpl22"

CDS complement(102865..103218)

/gene="rpl22"

/codon_start=1

/transl_table=11

/product="ribosomal protein L22"

/translation="MSAIKSLEAKAFSRYVRMSPFKVRRILDQIRGRSYEEALMILEF

MPYSGCKPVLKVLCSAASNAKHRFGTPKSSLFVCEARADKGPIMKRFRPRAQGRGFPI

QKPTCHIVITVSESS"

gene complement(103275..103553)

/gene="rps19"

CDS complement(103275..103553)

/gene="rps19"

/codon_start=1

/transl_table=11

/product="ribosomal protein S19"

/translation="MSRSIKKGPFVADHLLKKIEKMDSGGKRSVIITWSRASTVLPVM

IGHTIAVYNGQEHIPVLISDQMVGHKLGEFSPTRTYRGHVKSDKKSKR"

gene complement(103639..104472)

/gene="rpl2"

CDS complement(103639..104472)

/gene="rpl2"

/codon_start=1

/transl_table=11

/product="ribosomal protein L2"

/translation="MGIRFYKARTSGTRNRSVSDFSEITKSKPEKSLTYYKHRARGRN

NRGVITSRHRGGGHKRLYRQIDFKRNKLGVLAKVYSVEYDPNRNARIALLHYEDGEKR

YILQPNGLSVGDKVISNFDVPIQIGNAAPLKNIPLGTEVHNIELQPGKGGQLARAAGS

LAQIVAKEGNYVTLRIPSGEVRLIEKNCWATIGQIGNIEASNIVIGKAGRKRWLGKTP

KVRGVVMNPCDHAHGGGEGRSPIGRARPVTPWGRPALGQKTRKAKRYSNAFVIRSRKS

S"

CDS complement(104715..104993)

/codon_start=1

/transl_table=11

/product="ribosomal protein L23"

/translation="MIDLLKYPVLTEKATRLLERNQYTFDVDLRLNKTVIKSTIEELF

KVKVIAVNTYIPPRKKRRLGRFAGFKPAYKRVILTLKSDDSIPLFPES"

gene complement(105141..107342)

/gene="rbcL"

CDS complement(join(105141..106278,107053..107342))

/gene="rbcL"

/codon_start=1

/transl_table=11

/product="RuBisC/O large subunit"

/translation="MSPQTETKTGAGFKAGVKDYRLTYYTPEYQIAETDILAAFRMTP

QPGVPAEECGAAVAAESSTGTWTTVWTDGLTQLDRYKGRCYDLEPVPGEENQYIAYIA

YPIDLFEEGSVTNLLTSIVGNVFGFKALRSLRLEDLRIPPAYSKTFAGPPHGIQAERD

RLNKYGRAMLGCTIKPKLGLSAKNYGRAVYECLRGGLDFTKDDENVNSQSFMRWRDRF

MFCAEAIYKAQTETGEVKGHYLNVTAGTCEEMYKRAEYAAEIGVPIIMHDYITGGFTS

NTSLALYCRDNGLLLHIHRAMHAVIDRQRNHGIHFRVLAKTLRMSGGDHLHSGTVVGK

LEGEREVTLGFVDLMRDPYVEKDRSRGIYFTQDWCGMGGTMPVASGGIHVWHMPALVE

IFGDDACLQFGGGTLGHPWGNAPGAVANRVALEACVQARNEGRDLAREGGDVIRAACK

WSPELASACEVWKEIKFEFETIDKL"

exon complement(105141..106278)

/gene="rbcL"

/number=2

intron complement(106279..107052)

/gene="rbcL"

/number=1

misc_feature complement(106844..106864)

/gene="rbcL"

/note="3' motif for potential twintron"

exon complement(107053..107342)

/gene="rbcL"

/number=1

gene complement(107587..109996)

/gene="atpE"

CDS complement(join(107587..107820,108650..108687,

109861..109996))

/gene="atpE"

/codon_start=1

/transl_table=11

/product="ATPase epsilon subunit"

/translation="MSLEVSIIVPDRVFWKNDAQEIIFPTLTGQMGVLENHIPLLTGL

DTGVMKVRQGENWIAVAVMGGFALVNENKVTILVNEAVVSSDIDAQEAEAMYLQTKSV

FEKAAEGSKEKSEANVQYKKALARFEVTKELVI"

exon complement(107587..107820)

/gene="atpE"

/number=3

intron complement(107821..108649)

/gene="atpE"

/number=2

exon complement(108650..108687)

/gene="atpE"

/number=2

intron complement(108688..109860)

/gene="atpE"

/number=1

misc_feature complement(109056..109075)

/gene="atpE"

/note="3' motif for potential twintron"

misc_feature complement(109102..109124)

/gene="atpE"

/note="3' motif for potential twintron"

exon complement(109861..109996)

/gene="atpE"

/number=1

gene complement(110042..114641)

/gene="atpB"

CDS complement(join(110042..110641,111433..111545,

112333..112804,113581..113768,114572..114641))

/gene="atpB"

/codon_start=1

/transl_table=11

/product="ATPase beta subunit"

/translation="MAATVNTSAGVITQIIGPVMDIAFPPGSMPSIYNSLIIEGKIES

GENVKVVCEVQQLLGDNLVRAISMSATDGLQRGMSVVDTGSALSVPVGETTLGRIFNV

LGEPVDEMGDIDLSNKLPIHRSAPSFVDLDTQLAIFETGIKVVDLLAPYRRGGKIGLF

GGAGVGKTVLIMELINNIAKAHGGVSVFGGVGERTREGNDLYQEMKESGVINASNFKE

SKVALVYGQMNEPPGARMRVGLTALTMAEYFRDVNNQDVLLFIDNIFRFVQAGSEVSA

LLGRMPSAVGYQPTLATEMGGLQERITSTKEGSITSIQAVYVPADDLTDPAPATTFAH

LDATTVLSRNLAAKGIYPAVDPLDSTSTMLQPWIVGEEHYDIAQAVKRTLQRYKELQD

IIAILGLDELSEEDRLLVARARKVERFLSQPFFVAEVFTGSPGKYVSLAETIEGFKMI

LAGELDTLPEQAFYLVGALEEAVTKAASLS"

exon complement(110042..110641)

/gene="atpB"

/number=5

intron complement(110642..111432)

/gene="atpB"

/number=4

exon complement(111433..111545)

/gene="atpB"

/number=4

intron complement(111546..112332)

/gene="atpB"

/number=3

misc_feature complement(112174..112198)

/gene="atpB"

/note="3' motif for potential twintron"

exon complement(112333..112804)

/gene="atpB"

/number=3

intron complement(112805..113580)

/gene="atpB"

/number=2

misc_feature complement(113334..113356)

/gene="atpB"

/note="3' motif for potential twintron"

exon complement(113581..113768)

/gene="atpB"

/number=2

intron complement(113769..114571)

/gene="atpB"

/number=1

misc_feature complement(113978..114001)

/gene="atpB"

/note="3' motif for potential twintron"

exon complement(114572..114641)

/gene="atpB"

/number=1

CDS complement(join(114729..115214,119343..119481,

121218..121240))

/codon_start=1

/transl_table=11

/product="cytochrome b6/7 complex subunit V"

/translation="MSKVYDWFEERLEIQSIADDISSKYVPPHVNIFYCLGGLTFTCF

IIQVATGFAMTFYYRPTVTEAFASVQYIMNDVNFGWLIRSIHRWSASMMVLMMILHVC

RVYLTGGFKKPRELTWVTGVALASLTVSFGVTGYSLPWDQVGYWAVKIVTGVPDAIPV

IGSFVVELLRGSVSVGQSTLTRFYSLHTFVLPLLTATFMLAHFLMIRKQGISGPL"

exon complement(114729..115214)

/gene="petB"

/number=3

intron complement(115215..119342)

/gene="petB"

/number=2

misc_feature complement(115601..115620)

/note="3' motif for potential twintron"

misc_feature complement(116489..116513)

/note="3' motif for potential twintron"

CDS complement(116501..117430)

/note="group II intron reverse transcriptase/maturase K"

/codon_start=1

/transl_table=11

/product="putative reverse transcriptase and intron

maturase"

/translation="MFKYKDKSSSFFRFFKKNSLLYLLYDIYYFEFDKFLVQIFSSYD

PEFLENSTIVDHREFSTLRFLHYLYLRNWDQSFKTRISAVKYIKPLRNVNSMLSRNLL

SSIKFFYCRYLTHWIVLFPASVDISKIKSWILEYMISNINANTVSRTCKEISVLTGFS

FLGYSVKFSKTMAFNTPYILTSLKIYPDKFCILRSLLTKSVISRENFFPIAKRSWVNF

SDYDIVLNFRRIILDLVYYYKYSYSLKSLHFVLYLLKFSCAKTLACKNRITLTQVFNK

YGSVLTTEKRIYMGDYHRVYSISFPLLSEFKKI"

misc_feature complement(116874..116895)

/note="3' motif for potential twintron"

CDS complement(117286..118011)

/codon_start=1

/transl_table=11

/product="hypothetical protein"

/translation="MCKKTCSYSSLGANKLATHLCSPYFFLEVLSSFSKISQNEMSLY

SHYNTRLKFISHLHAESLSQQFRSYNYVFYTSGKKRKLGSVNLALTDSFSKHREFIVN

ESLYLILKFIFEPKFESWKQMDSSSHILTSCGYSIQEIIPNFCSHIYRRSTNYLGING

ELPFIDPAICLLLLRRYIKDHKFLTLINNFLISSCSNIRINQVPFLDSSKKIVCCIYC

TIFIILNLINFLFRFLVVMILNF"

misc_feature complement(118299..118321)

/note="3' motif for potential twintron"

exon complement(119343..119481)

/gene="petB"

/number=2

intron complement(119482..121217)

/gene="petB"

/number=1

exon complement(121218..121240)

/gene="petB"

/number=1

gene 121520..121654

/gene="psbN"

CDS 121520..121654

/gene="psbN"

/codon_start=1

/transl_table=11

/product="photosystem II N protein"

/translation="METPAFFSAIFLGCLLLSITGYSIYIGFGPASKELRDPFEEHED

"

gene 121715..121951

/gene="psbH"

CDS 121715..121951

/gene="psbH"

/codon_start=1

/transl_table=11

/product="photosystem II 10 kDa protein"

/translation="MATTSKTKTPITQGKVTALGSALKPLNSEYGKVAPGWGTTVVMA

VFMALFAVFLVIILEIYNSSVILDNVGMSWKSLG"

gene complement(122158..122750)

/gene="psbT"

CDS complement(join(122158..122228,122726..122750))

/gene="psbT"

/codon_start=1

/transl_table=11

/product="photosystem II T protein"

/translation="MEALVYTFLLIATLGIIFFAIFFREPPRIVK"

exon complement(122158..122228)

/gene="psbT"

/number=2

intron complement(122229..122725)

/gene="psbT"

/number=1

misc_feature complement(122415..122434)

/gene="psbT"

/note="3' motif for potential twintron"

misc_feature complement(122526..122547)

/gene="psbT"

/note="3' motif for potential twintron"

exon complement(122726..122750)

/gene="psbT"

/number=1

gene complement(122872..126010)

/gene="psbB"

CDS complement(join(122872..123386,124162..125153,

125991..126010))

/gene="psbB"

/codon_start=1

/transl_table=11

/product="photosystem II CP47 chlorophyll apoprotein"

/translation="MGLPWYRVHTVVLNDPGRLIAVHLMHTALVSGWAGSMALYEIAI

FDPSDPVLNPMWRQGMFVLPFMTRLGVTKSWGAWSIDGGNAANPGFWSYEGVALAHIV

LSGLLFLAAIWHWVYWDLQLFRDPRTGDSALDLPKIFGIHLFLSGLLCFGFGAFHVTG

LFGPGIWVSDPYGITGSIQPVTPAWGAEGFDPYNPGGIASHHIAAGILGIIAGLFHLT

VRPPQRLYKVLRMGNIETVLSSSIAAVFWSAFVVAGTMWYGSAATPIELFGPTRYQWD

QSFFQEETQRRVQKSLEEGSTLSQAWAKIPEKLAFYDYIGNNPAKGGLFRSGPMDNGD

GIAAGWLGHAVFTDKSGRELTVRRMPTFFETFPVLLLDQDGIVRADVPFRRAESKYSI

EQVGVSVSFYGGELDGVSFNDPATVKKYARRSQLGEIFEFDRATLQSDGVFRSSPRGW

FTFGHLCFALLFFFGHIWHGARTIFRDVFAGIDPDLEEQIEFGAFQKLGDVTTRKQAV

"

exon complement(122872..123386)

/gene="psbB"

/number=3

intron complement(123387..124161)

/gene="psbB"

/number=2

misc_feature complement(124003..124027)

/gene="psbB"

/note="3' motif for potential twintron"

exon complement(124162..125153)

/gene="psbB"

/number=2

intron complement(125154..125990)

/gene="psbB"

/number=1

misc_feature complement(125725..125749)

/gene="psbB"

/note="3' motif for potential twintron"

misc_feature complement(125726..125748)

/gene="psbB"

/note="3' motif for potential twintron"

exon complement(125991..126010)

/gene="psbB"

/number=1

gene 126168..126239

/gene="trnN"

tRNA 126168..126239

/gene="trnN"

/product="tRNA-Asn"

/anticodon=(pos:126200..126202,aa:Asn,seq:gtt)

gene 126261..126334

/gene="trnR"

tRNA 126261..126334

/gene="trnR"

/product="tRNA-Arg"

/anticodon=(pos:126295..126297,aa:Arg,seq:acg)

gene complement(126565..126646)

/gene="trnL"

tRNA complement(126565..126646)

/gene="trnL"

/product="tRNA-Leu"

/anticodon=(pos:complement(126610..126612),aa:Leu,seq:tag)

gene complement(126969..130530)

/gene="psaC"

CDS complement(join(126969..127102,127722..127746,

128574..128587,129882..129912,130489..130530))

/gene="psaC"

/codon_start=1

/transl_table=11

/product="photosystem I subunit VII"

/translation="MSHSVKIYDTCIGCTQCVRACPTDVLEMVPWDGCKAGQIASAPR

TEDCVGCKRCESACPTDFLSVRVYLGSETTRSMGLAY"

exon complement(126969..127102)

/gene="psaC"

/number=5

intron complement(127103..127721)

/gene="psaC"

/number=4

exon complement(127722..127746)

/gene="psaC"

/number=4

intron complement(127747..128573)

/gene="psaC"

/number=3

exon complement(128574..128587)

/gene="psaC"

/number=3

intron complement(128588..129881)

/gene="psaC"

/number=2

exon complement(129882..129912)

/gene="psaC"

/number=2

intron complement(129913..130488)

/gene="psaC"

/number=1

exon complement(130489..130530)

/gene="psaC"

/number=1
